# αβ,α′β′-Diepoxyketones are mechanism-based inhibitors of nucleophilic cysteine enzymes†

**DOI:** 10.1039/d3cc02932h

**Published:** 2023-10-02

**Authors:** Mariska de Munnik, Jasper Lithgow, Lennart Brewitz, Kirsten E. Christensen, Robert H. Bates, Beatriz Rodriguez-Miquel, Christopher J. Schofield

**Affiliations:** a Chemistry Research Laboratory, Department of Chemistry and the Ineos Oxford Institute of Antimicrobial Research, University of Oxford 12 Mansfield Road Oxford OX1 3TA UK christopher.schofield@chem.ox.ac.uk; b Chemical Crystallography, Chemistry Research Laboratory, Department of Chemistry, University of Oxford 12 Mansfield Road Oxford OX1 3TA UK; c Tres Cantos Medicines Development Campus, GlaxoSmithKline Calle Severo Ochoa 2, Tres Cantos Madrid Spain

## Abstract

Epoxides are an established class of electrophilic alkylating agents that react with nucleophilic protein residues. We report αβ,α′β′-diepoxyketones (DEKs) as a new type of mechanism-based inhibitors of nucleophilic cysteine enzymes. Studies with the l,d-transpeptidase Ldt_Mt2_ from *Mycobacterium tuberculosis* and the main protease from SARS-CoV-2 (M^pro^) reveal that following epoxide ring opening by a nucleophilic cysteine, further reactions can occur, leading to irreversible alkylation.

## Abstract 2

αβ,α′β′-Diepoxyketones (DEKs) inhibit nucleophilic cysteine enzymes. DEKs react with a transpeptidase and the SARS-CoV-2 main protease *via* epoxide opening; retro-aldol and other reactions can then occur, enabling irreversible alkylation.

## Graphical abstract

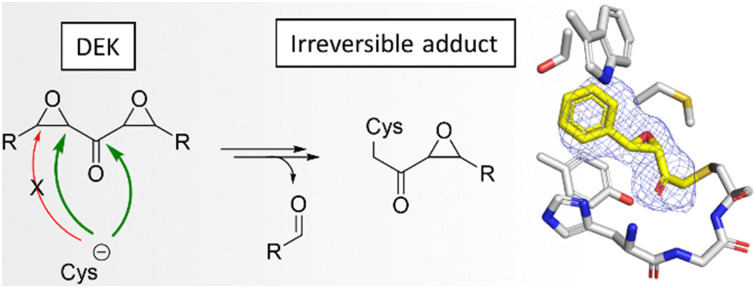

Most covalently reacting enzyme inhibitors bear an electrophilic functional group that reacts with a nucleophile to enable covalent protein modification.^1^ Although many such inhibitors work by apparently simple acylation, alkylation or conjugate addition reactions, some undergo further reaction after initial covalent modification. Such mechanism-based inhibitors can be found in drugs,^2–4^ with one such example being inhibitors of the nucleophilic serine-β-lactamases, such as clavulanic acid.^5,6^

Despite the long-standing importance of covalently reacting drugs, concerns regarding potential toxicity have hindered their development. Covalently reacting drugs are, however, the subject of recent renewed interest,^1,7^ and are currently the basis for multiple drug development programs, including in oncology and antimicrobials.^8–11^ Covalent targeting of a prevalent oncogenic mutation in K-Ras (K-Ras^G12C^) has led to development of sotorasib and adagrasib.^12^ Various medicinal chemistry programs targeting the main protease (M^pro^) of SARS-CoV-2 have focussed on covalent reaction of the catalytic cysteine residue, with nirmatrelvir, a reversibly reacting nitrile-bearing inhibitor, being approved for COVID-19 treatment.^13,14^ The L,D-transpeptidase Ldt_Mt2_ of *Mycobacterium tuberculosis*, which is a target for TB treatment,^15^ is amenable to covalent inhibition *via* reaction with its catalytic cysteine.^16–18^

Epoxides are an established class of electrophilic alkylating agents, and are used to inhibit nucleophilic cysteine (and serine) proteases.^1,19,20^ Many epoxide inhibitors of cysteine or serine proteases contain peptide backbones, *e.g.* proteasome inhibitors,^21–24^ though the small molecule epoxide fosfomycin is a clinically important antibiotic (Fig. 1A).^25,26^

**Fig. 1:**
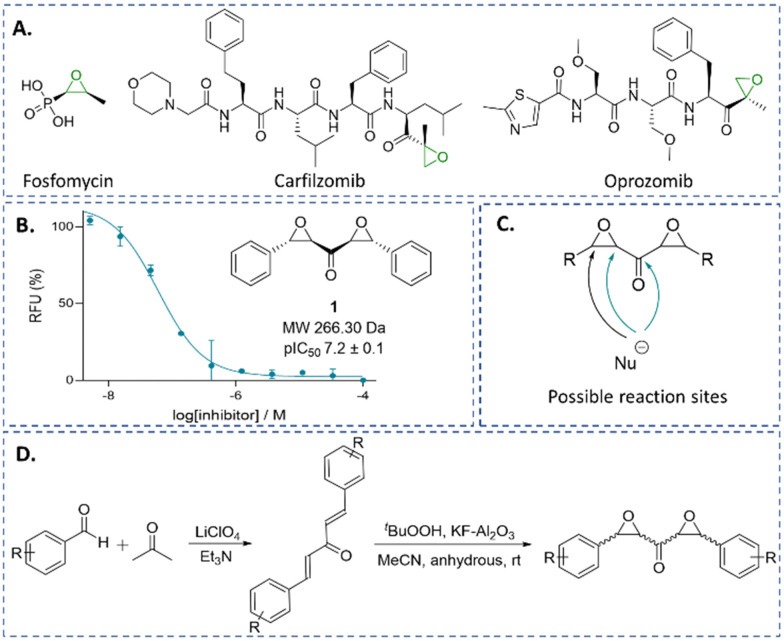
αβ,α′β′-Diepoxyketones (DEKs) react with nucleophilic cysteine enzymes. (A) Examples of epoxide-bearing drugs. (B) DEK 1 was identified as a potent inhibitor of Ldt_Mt2_. (C) Symmetrical DEKs contain 3 potential sites for interactions with nucleophiles, as well as three oxygens that may react with electrophiles. Arrows in teal represent pathways consistent with mechanistic studies. (D) Synthesis of DEKs 1 and 4–11.

We are interested in identifying new types of covalently reacting modulators of biological function. Recently, we reported on a high-throughput screen aiming to identify new electrophilic inhibitors of Ldt_Mt2_ and other nucleophilic enzymes.^27^ Here, we describe the identification of the small molecule *trans*,*trans* αβ,α′β′-diepoxyketone (DEK) 1 (Fig. 1B), and the potency and mechanism of 1 and related DEKs 4–12 for Ldt_Mt2_ and SARS-CoV-2 M^pro^ inhibition; the results reveal DEKs as a mechanistically interesting class of electrophile.

Symmetrical DEKs have 3 obvious positions that may react with nucleophiles and have potential to undergo further reactions (Fig. 1C). DEK 1 exhibited potent inhibition of Ldt_Mt2_, with a pIC_50_ of 7.2 ± 0.1, with 30 min pre-incubation (Fig. 1B). To investigate the mode of reaction of 1 with Ldt_Mt2_, we carried out protein-observed mass spectrometry employing solid-phase extraction (SPE-MS). The results reveal that 1 covalently reacts with Ldt_Mt2_, giving an initial adduct (2) with a +267 Da mass shift relative to unmodified Ldt_Mt2_ (Fig. 2B and Table S1, ESI†), corresponding to addition of one molecule of 1 to Ldt_Mt2_, which has a single cysteine (Cys354). This adduct (2) was transient, converting within 2 h into one with a mass shift of +160 Da relative to unmodified Ldt_Mt2_, provisionally assigned as 3. We proposed the reaction involves nucleophilic attack of Cys354 on the carbonyl-group adjacent carbon of one of the symmetrical epoxides, with ring opening to form 2, followed by retro-aldol fragmentation, releasing benzaldehyde (Fig. 2C). Alternatively, the reaction may proceed though reaction at the carbonyl carbon to generate a hemithioketal, after which rearrangement may occur (Fig. 2C).^28^

**Fig. 2:**
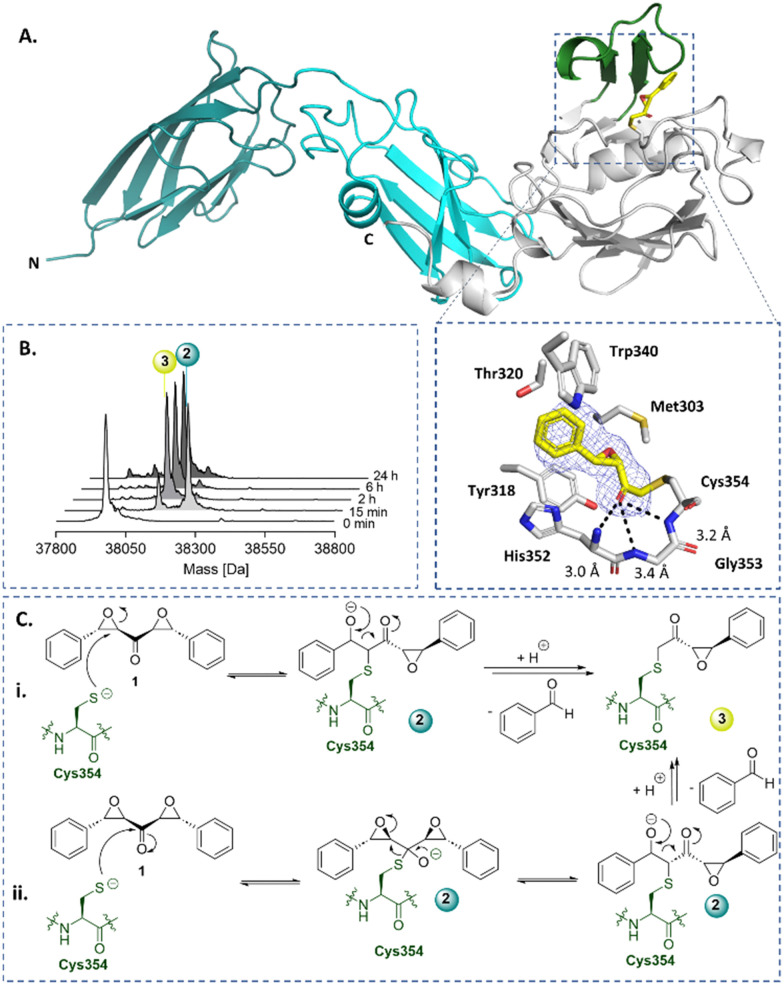
X-ray crystallography and protein-observed SPE-MS studies inform on the mechanism of DEK inhibition. (A) Views from a crystal structure derived by reaction of Ldt_Mt2_ with DEK 1 (yellow, PDB: 8BK3). The immunoglobulin-like domains are in teal and cyan. The catalytic domain is grey, with the active site lid in green. The mF_o_-DF_c_ polder OMIT map is contoured at 3.0*σ*, carved around Cys354 bound 1 (refined as 3) and shown in blue mesh. Polar interactions are shown in black dashes. (B) Protein-observed SPE-MS experiments inform on the mechanism of reaction of 1 (20 μM) with Ldt_Mt2_ (1 μM). (C) The proposed mechanisms for reaction of Cys354 of Ldt_Mt2_ (in green) with 1*via* reaction with (i) the carbonyl adjacent carbon or (ii) the carbonyl carbon, followed by retro-aldol fragmentation.

The identity of 3 was validated by X-ray crystallography, using reported conditions,^27^ wherein 1 was introduced through soaking; a structure of Ldt_Mt2_ reacted with 1 was obtained (2.15 Å resolution, *P*12_1_1 space group, PDB: 8BK3, Table S2, ESI†). As reported, Ldt_Mt2_ crystallised with two molecules (chains A and B) in the asymmetric unit. While this structure manifested clear additional electron density at the chain A active site, only partial density was observed at that of chain B, thus inhibitor modelling was only performed in chain A. The additional electron density in chain A supports the proposed structure of adduct 3 (Fig. 2). The carbonyl of 3 projects into the proposed oxyanion hole, formed by the backbone NH groups of His352, Gly353 and Cys354 (distances of 3.0 Å, 3.4 Å and 3.2 Å, respectively).^29^ Extensive hydrophobic interactions of 3 with active site residues Tyr318, His352, Trp340, Thr320, and Met303 were observed.

In aqueous solution, 1 was found to be stable for at least 12 h (Fig. S3, ESI†). Cysteine reacted with 1, apparently yielding a product analogous to adduct 3 (Fig. S4, ESI†). No evidence for reaction of 1 with serine, lysine, threonine, tyrosine, arginine, or histidine was observed by ^1^H NMR or LCMS under the tested conditions (Fig. S5, ESI†).

To further analyse the inhibitory potency and mechanism of the DEKs, we prepared derivatives of 1. Synthesis involved preparation of the diene ketones *via* solvent-free aldol condensation, mediated by lithium perchlorate and Et_3_N,^30^ followed by epoxidation using ^*t*^BuOOH and KF-Al_2_O_3_,^31,32^ to yield stereo-isomeric mixtures of DEKs 1 and 4–11 (Fig. 1D and Table S3, ESI†).

No substantial difference in inhibition between diastereomerically pure 1 and enantiomerically pure 1 was observed. While we did not obtain the pure *cis*,*cis* diastereomer of 1, a diastereomeric mixture of 1 (∼1 : 3 ratio of *trans*,*trans* : *cis*,*cis* stereoisomers) manifested potent, but decreased, Ldt_Mt2_ inhibition compared to diastereomerically pure 1 (pIC_50_ 5.6 ± 0.04 compared to 6.2 ± 0.07 for diastereomerically pure 1, with 15 min preincubation, Fig. S6, ESI†). The results imply the importance of the *trans,trans* stereochemistry for potent Ldt_Mt2_ inhibition by the DEKs. Recrystallisation of diastereomeric mixtures from ethanol afforded the corresponding pure *trans*,*trans* diastereomers, as supported by ^1^H NMR analysis and small molecule X-ray diffraction (Table S4, ESI†), except for DEKs 5 and 8, which were tested as diastereomeric mixtures (*trans*,*trans*:*cis*,*cis* ratio ∼2 : 1 and ∼1.2 : 1, respectively).

Dose–response assays of 4–11 with Ldt_Mt2_ showed decreased potency compared to 1 (Table S3 and Fig. S1, ESI†). Determination of the second-order rate constants for covalent target inactivation (*k*_inact_/*K*_I_)^33^ for Ldt_Mt2_ manifested the highest rate of inhibition for 1 (*k*_inact_/*K*_I_ of 484.3 ± 28.4 M^−1^ s^−1^, Table S3 and Fig. S7, ESI†). DEKs 5–7 and 9 were observed to inhibit Ldt_Mt2_, while no evidence for inhibition was observed with 4, 8 and 10. The kinetic rate constant for reactivity with GSH (*k*_chem_)^27,34^ was found to be below the assay limit for all DEKs (*k*_chem_ of <0.08 M^−1^ s^−1^ and half-life (*t*_1/2_) >8.7 h), except 7 and 8 (*k*_chem_ of 1.71 ± 0.24 and 1.11 ± 0.20 M^−1^ s^−1^, and *t*_1/2_ of 24 min and 38 min, respectively; Table S3 and Fig. S8, ESI†). DEKs therefore apparently exhibit lower intrinsic reactivity compared to the common cysteine reactive acrylate, maleimide and isothiocyanate groups (*t*_1/2_ < 1.0 min), and, with the exceptions of 7 and 8, chloroacetamide (5.8 h).^35^ MS studies of the reaction of GSH and 1 manifested an adduct analogous to 3 (Fig. S9, ESI†).

Protein-observed SPE-MS assays of 4–11 demonstrated covalent modification of Ldt_Mt2_ with 4–10, which manifested adducts analogous to those with 1 (Fig. S2 and Table S1, ESI†) supporting the generality of the proposed mechanism. Additional peaks of +18 Da were observed with both unfragmented and fragmented adducts of 4–10, likely due to ring opening of the second epoxide (Fig. S10, ESI†). With 1, 5, 6 and 7, over 24 h, a second fragment adduct was observed with a +56 mass shift relative to the unmodified enzyme (Fig. S10, ESI†).

DEK 1 apparently displayed a low level of β-elimination of the reacted Cys354 residue, likely to form a dehydroalanine residue (Dha, ∼5% in 24 h, as evidenced by a −34 Da mass shift relative to unmodified Ldt_Mt2_, Fig. S2 and S10, ESI†).^36–38^ Interestingly, the *ortho*-trifluoromethoxy substituents on the phenyl groups of 5 promoted Dha formation (∼30% in 24 h). Dha formation was additionally observed following reaction with 4 (∼2.5% in 24 h) and 7 (∼16% in 24 h). In the cases of 6 and 8–10, no evidence for Dha formation was observed.

While inhibition assays with the α,β-monoepoxyketone 12 did not manifest inhibition of Ldt_Mt2_, protein-observed SPE-MS assays of Ldt_Mt2_ (1 μM) with 12 (100 μM) evidenced covalent reaction. As with DEKs 1 and 4–10, initial measurements (2 h) showed the most abundant adduct to have a mass shift of +224 Da, corresponding to the addition of a single molecule of 12. A +119 Da adduct was observed to become abundant after 6 h (Fig. S2, ESI†), indicating that the retro-aldol fragmentation is conserved between mono- and diepoxide derivatives.

While Ldt_Mt2_ contains only a single cysteine, in principle, the DEKs may alkylate other nucleophilic residues.^39,40^ To investigate whether the DEKs react selectively with Cys-354 of Ldt_Mt2_, we performed protein-observed SPE-MS assays with Ldt_Mt2_ that had been preincubated with ebselen, which is known to selectively and irreversibly react with Cys354.^16^ When 1 and 4–10 were combined with the Ldt_Mt2_-ebselen complex, no reaction was observed, evidencing that inhibition arises from at least partially, selective reaction with Cys354 (Fig. S11, ESI†).

To further investigate the reactivity of DEKs with nucleophilic cysteine enzymes, dose–response assays of 1 and 4–11 were performed with SARS-CoV-2 M^pro^;^41,42^ note that the covalent reaction of SARS-CoV M^pro^ with epoxides has been reported.^43^ While DEKs 1 and 9 were inhibitors of M^pro^ (pIC_50_ values of 4.6 ± 0.3 and 5.9 ± 0.2, respectively), no inhibition was observed with 4–8 and 10–11 (Table S3 and Fig. S12, ESI†), providing further evidence for potential of the DEKs to react selectively.

Protein-observed SPE-MS experiments with M^pro^ and the DEKs 1 and 9 (Fig. S13, ESI†) manifested a +266 Da adduct (analogous to species 2, Fig. 2C), with a +160 Da adduct (analogous to species 3) becoming apparent over time. A second molecule of 1 was observed to bind to M^pro^ after 3 h (as evidenced by a mass shift of +266 Da relative to the +160 adduct), indicating reaction with a second residue, likely with one or more of the 12 cysteine residues of M^pro^. Notably, the second adduct did not fragment by retro-aldol reaction, implying that this pathway can be promoted by the active site, likely by binding of one of the DEK-derived oxygens in the oxyanion hole of M^pro^.^44^ Incubation of M^pro^ with 9 resulted in a single adduct of +186 Da, which can be assigned to a fragmented species analogous to species 3 (Fig. 2C).

As epoxide-bearing compounds may inhibit serine proteases, notably including proteasomes,^45,46^ we tested the ability of the DEKs to inhibit the nucleophilic serine enzyme BlaC, a class A β-lactamase of *M. tuberculosis*. None of compounds 1 and 4–12 exhibited inhibitory potency for BlaC (Fig. S14, ESI†).

The combined results of the reaction of DEKs with GSH, cysteine, Ldt_Mt2_ and SARS-CoV-2 M^pro^, imply a conserved reaction mechanism, involving epoxide opening followed by retro-aldol reaction. Importantly, the results reveal different reactivity of the 12 M^pro^ cysteine residues with DEKs, indicating that selectivity for some proteins should be achievable; note that previous results showed that excess ebselen reacts covalently with all 12 cysteine residues.^47^

The results identify DEKs as a new class of nucleophilic cysteine reacting covalent ligands. Variations on the DEK functionality can be readily envisaged *e.g.*, by substituting one or both epoxides for other covalently reacting electrophiles, such as aziridines or acylating agents. Notably, some natural products contain more than one epoxide, sometimes in a contiguous arrangement,^48^ though to our knowledge the DEK functional group has not been identified in natural products. Interestingly, DEKs have 5 hypothetical sites for reaction with nucleophiles (Fig. 1C), and they hold potential for subsequent addition of a second nucleophile. This could be useful in enabling (i) formation of cross-linked enzyme-inhibitor complexes (as can occur with other mechanism based inhibitors, *e.g.*, certain β-lactamase inhibitors),^49^ (ii) labelling of an inhibited protein for analytical or diagnostic purposes, (iii) the capture of enzyme substrates, and (iv) covalent gluing of protein–protein interactions; note that epoxides are used in commonly used polyepoxide glues.^50^ The ability of DEKs to fragment after initial covalent reaction might be useful in releasing a functional fragment, *e.g.*, a cytotoxic agent (the cytotoxicity of benzaldehyde in tumour cells has been reported^51^).

We are very grateful to Eidarus Salah for SARS-CoV-2 M^pro^. We thank the Department of Biochemistry (Oxford) for the use of the 950 MHz spectrometer and Dr Patrick Rabe supporting NMR experiments. The project was co-funded by the Tres Cantos Open Lab Foundation (Project TC241 and project TC297). It was supported by funding from the Biotechnology and Biological Sciences Research Council (BBSRC) [BB/M011224/1] and the Wellcome Trust (106244/Z/14/Z).

## Conflicts of interest

There are no conflicts to declare.

## Supplementary Material

CC-059-D3CC02932H-s001

CC-059-D3CC02932H-s002
