# Supplementary material for: αβ,α′β′-Diepoxyketones are mechanism-based inhibitors of nucleophilic cysteine enzymes
Source: Chem Commun (Camb). 2023 Oct 2;59(86):12859–62. doi: 10.1039/d3cc02932h (PMC10601815; doi:10.1039/d3cc02932h)
Supplement: CC-059-D3CC02932H-s001 [file CC-059-D3CC02932H-s001.pdf]

Electronic Supplementary Material (ESI) for ChemComm.
This journal is © The Royal Society of Chemistry 2023

Supplementary information

αβ,α′β′-Diepoxyketones are Mechanism-Based Inhibitors of Nucleophilic Cysteine Enzymes

Mariska de Munnik[a], Jasper Lithgow[a], Lennart Brewitz[a], Kirsten E. Christensen[b], Robert Bates[c], Beatriz Rodriguez-Miquel[c], and Christopher J. Schofield[*a]

[a] Chemistry Research Laboratory, Department of Chemistry and the Ineos Oxford Institute of Antimicrobial Research, University of Oxford, 12 Mansfield Road, Oxford, OX1 3TA, United Kingdom.

[b] Chemical Crystallography, Chemistry Research Laboratory, Department of Chemistry, University of Oxford, 12 Mansfield Road, Oxford, OX1 3TA, United Kingdom.

[c] Tres Cantos Medicines Development Campus GlaxoSmithKline, Calle Severo Ochoa 2, Tres Cantos, Madrid, Spain.

[*] Email: christopher.schofield@chem.ox.ac.uk

Experimental details

Materials

Recombinant Ldt$_{Mt2}$ was produced in *Escherichia coli* and purified (>95% purity by SDS-PAGE analysis) as reported.[1] Recombinant SARS-CoV-2 M^{pro} was produced by Eidarus Salah and purified as reported.[2] Probe 1 (2-(6-(((2,4-dinitrophenyl)sulfonyl)oxy)-3-oxo-3H-xanthen-9-yl)benzoic acid) and FC-5 was synthesised as reported.[3, 4] DEK **1** was initially obtained from the GSK compound collection in enantiopure form, and then synthesised as a single diastereomer as outlined below. Epoxide **12** was purchased from Enamine in diastereomerically pure from. All reagents for the preparation of **1** and **4** – **11** were from commercial sources (Sigma-Aldrich, Inc.; Fluorochem Ltd; Alfa Aesar; Manchester Organics) and were used as received. Purifications by column chromatography were performed using an automated Biotage® Selekt instrument (wavelengths monitored: 254 and 280 nm) equipped with pre-packed Biotage® Sfär Silica D chromatography cartridges. Thin layer chromatography (TLC) was carried out using Merck Silica gel 60 F254 TLC plates. Melting points (m.p.) were determined using a Stuart SMP-40 automated melting point apparatus. Infrared (IR) spectroscopy was performed using a Bruker Tensor-27 Fourier transform infrared (FT-IR) spectrometer. High-resolution mass spectrometry (HRMS) was performed using electro-spray ionization (ESI) mass spectrometry (MS) in the positive ionisation mode employing a Thermo Scientific Exactive mass spectrometer (ThermoFisher Scientific). Nuclear magnetic resonance (NMR) spectroscopy was performed using a Bruker AVANCE AVIIIHD 400 instrument.

Inhibition studies

Ldt$_{Mt2}$ fluorogenic assays were performed as described.[3] Ldt$_{Mt2}$ (100 nM) was incubated with varying concentrations of a potential inhibitor (400 µM – 20.3 nM) for 10 min in the assay buffer (50 mM HEPES, pH 7.2, 0.01% (*v*/*v*) Triton X-100) and then assayed using Probe 1 (25 µM).

SARS-CoV-2 M^{pro} inhibition assays were performed as described.[5] M^{pro} (150 nM) was incubated with varying concentrations of inhibitor (100 µM – 1.7 nM) for 15 min in assay buffer (20 mM HEPES, pH 7.5, 50 mM NaCl) and then assayed using a 37mer peptide as the substrate (ALNDFSNSGSDVLYQPPQTSITSAVLQSGFRKMAFPS-NH_2, 2 µM).

BlaC fluorogenic assays were performed as described.[4, 6] BlaC (14 nM) was incubated with varying concentrations of inhibitor (400 µM – 20.3 nM) for 10 min in assay buffer (100 mM sodium phosphate pH 7.5, 0.01% (*v*/*v*) Triton X-100) and then assayed using FC5 (10 µM).

The 'intrinsic' thiol reactivity (k_{chem}) was determined as described.[6] *L*-Glutathione (500 nM) was incubated with varying concentrations of inhibitor (400 µM – 20.3 nM) and Probe 1 (10 µM) for 15 h in assay buffer (50 mM HEPES, pH 7.2, 0.01% (*v*/*v*) Triton X-100).

The second-order rate constant of covalent target inactivation (k_{inact}/K_I) was determined as described.[6] Ldt_{Mt2} (100 nM) was incubated with varying concentrations of inhibitor (400 µM – 20.3 nM) and Probe 1 (10 µM) for 3.5 h in assay buffer (50 mM HEPES, pH 7.2, 0.01% (*v*/*v*) Triton X-100).

Protein observed SPE-MS assays

Protein-observed SPE-MS experiments with Ldt_{Mt2} were performed as described.[6] Ldt_{Mt2} (1 µM) in 50 mM tris, pH 7.5 was incubated with an inhibitor (20 µM) at room temperature.

SARS-CoV-2 M^{pro} protein-observed SPE-MS experiments were performed as described.[2, 5]

X-ray crystallography

Recombinant Ldt_{Mt2} (Δ1-55; with the N-terminal His_6-Tag removed, in 50 mM tris, pH 8.0, 100 mM NaCl) was crystallised using sitting drop vapor diffusion at 4 °C, according to a reported procedure.[6] The inhibitor was introduced to the crystals through soaking (1.5 mM, 24 h), after which time the crystals were cryocooled and stored in liquid nitrogen. Datasets were collected using the MX beamline I03 at the Diamond Light Source synchrotron (Harwell, United Kingdom). Structures were solved by molecular replacement using Phaser[7], using PDB entry 6RRM[8] as the search model. Alternating cycles of refinement using PHENIX[9] and manual model building using COOT[10] were performed until R_{work} and R_{Free} converged. Data collection and refinement statistics can be found in Table S2. Ligands were visualised by mF_o-DF_c polder OMIT map.[11]

Single crystal X-ray diffraction data were collected for **1** at 150 K using a (Rigaku) Oxford Diffraction SuperNova diffractometer and CrysAlisPro. The structure was solved using 'Superflip'[12] before refinement with CRYSTALS[13, 14] as described in the SI (CIF). The crystallographic data have been deposited with the Cambridge Crystallographic Data Centre (CCDC 2262059), and copies of these data can be obtained free of charge from The Cambridge Crystallographic Data Centre via www.ccdc.cam.ac.uk/data_request/cif.

Preparation of recombinant BlaC protein

A codon-optimised synthetic gene (GeneArt, Thermo Fisher Scientific) encoding for BlaC Δ1-40 was amplified and cloned into the expression vector pCold using Sal1-HF (New England BioLabs) and Not1-HF (New England BioLabs) digestion and ligation using T4 DNA ligase (New England BioLabs) according to the manufacturer's protocol. The ampicillin resistance gene of the vector was exchanged for the

kanamycin resistance gene using Gibson Assembly,[15] and transformed with *Escherichia coli* BL21(DE3). An overnight culture of *E. coli* BL21(DE3) pCold-BlaC Δ1-40 was grown at 37 °C at 180 rpm in 2xTY media (with 50 µg/mL kanamycin). This culture was used to inoculate fresh 2xTY media containing 50 µg/mL kanamycin (1% (*v/v*)), which was grown at 37 °C at 180 rpm to OD_{600} of 0.6. Then, 0.5 mM Isopropyl β-D-thiogalactopyranoside (IPTG) was added and the culture was incubated at 18 °C at 180 rpm for an additional 16 h. Cells were collected by centrifugation (11,000 x g, 8 min), and stored at -80 °C.

The cell pellet was resuspended in HisTrap Buffer A (25 mM Tris-HCl pH 8.0, 500 mM NaCl, 0.5 mM tris(2-carboxyethyl)phosphine) (TCEP), 5% (*v/v*) glycerol, 20 mM imidazole) in the presence of DNase I, and lysed using a Continuous Flow Cell Disruptor (Constant Systems, 20 kpsi). The lysates were centrifuged (32,000 x g, 20 min), passed through a 0.45 µm filter, and loaded onto a 5 mL HisTrap column (GE Life Sciences) that had been pre-equilibrated in HisTrap Buffer A. The column was washed with HisTrap Buffer A, followed by a gradient running from 0 % to 100 % (*v/v*) HisTrap Buffer B (25 mM Tris-HCl pH 8.0, 500 mM NaCl, 0.5 mM TCEP, 5% (*v/v*) glycerol, 250 mM imidazole). Fractions containing BlaC (as observed by SDS-PAGE) were combined, the buffer was exchanged to HisTrap Buffer A, and the HisTag was cleaved using recombinant 3C protease at 4 °C, over 12 h. The HisTag cleaved BlaC was passed through a 5 mL HisTrap column (GE Life Sciences) and washed with HisTrap Buffer A. The BlaC containing fractions (as observed by NanoDrop (Thermo Scientific) analysis) were concentrated and loaded onto a 300 mL Superdex 75 column (GE Life Sciences) pre-equilibrated in gel filtration buffer (25 mM Tris-HCl pH 8.0, 500 mM NaCl, 0.5 mM TCEP, 5% (*v/v*) glycerol). BlaC was eluted using the gel filtration buffer. Fractions containing BlaC (as observed by SDS-PAGE) were combined, concentrated, and frozen using liquid nitrogen. The identity and purity of BlaC was confirmed by mass spectrometry (calculated mass 28740 Da, observed deconvoluted mass 28740 Da) and SDS-PAGE (>95% purity).

Procedure for the synthesis of the diene precursors of 1 and 4-11 (General Procedure A)

A modified literature procedure was followed to prepare the αβ,α′β′-dienone precursors of **1** and **4-11,** i.e. **13**-**18**.[16] To a neat solution of lithium perchlorate ($LiClO_4$) (20 mmol, 2 equiv.), benzaldehyde (or a benzaldehyde derivative, as specified; 20 mmol, 2 equiv.), and the requisite ketone (10 mmol, 1 equiv.) was added triethylamine (Et_3N) (0.3 mL, 2 mmol, 0.1 equiv.). The mixture was stirred at room temperature (rt) and the reaction progress was monitored by thin layer chromatography (TLC). Upon completion of the reaction, a saturated aqueous ammonium chloride (NH_4Cl) solution was added, and the resulting mixture was extracted with dichloromethane. The organic extracts were dried over anhydrous sodium sulfate (Na_2SO_4) and concentrated under reduced pressure. The crude mixture was purified by flash column chromatography.

Procedure for the synthesis of DEKs 1 and 4-11 (General Procedure B)

A modified literature procedure was followed to prepare αβ,α′β′-diepoxide ketones **1** and **4**-**11**.[17] To a stirred suspension of potassium fluoride supported on alumina (KF-Al_2O_3) (prepared as described[18]) in *tert*-butyl hydroperoxide (^{t}BuOOH; 5.0-6.0 M in decane, 3.8 mL, ~21 mmol) under N_2 was added a solution of the specified αβ,α′β′-dienone (2 mmol) in anhydrous acetonitrile (10 mL). The mixture was stirred at rt, and the reaction progress was monitored by TLC. Upon completion of the reaction, the mixture was filtered with a sintered funnel *in vacuo*. The filtrate was washed with brine and extracted with ethyl acetate. The combined organic extracts were dried over anhydrous Na_2SO_4 and concentrated *in vacuo*. The crude residue was purified by recrystallisation from ethanol to afford the *trans/trans*-αβ,α′β′-diepoxide ketone.

(1*E*,4*E*)-1,5-Bis(4-(trifluoromethoxy)phenyl)penta-1,4-dien-3-one (13)

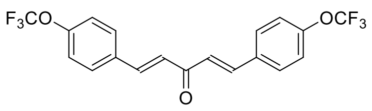

According to General Procedure A, diene **13** (2.37 g, 59%) was obtained from 4-(trifluoromethoxy)benzaldehyde (3.80 g, 20 mmol) and acetone (0.58 g, 10 mmol), following column chromatography [50 g Sfär Silica D; 120 mL/min, 100% cyclohexane (2 CV (column volumes)), followed by a linear gradient (12 CV): 0%→10% acetone in cyclohexane]. The analytical data for **13** were in agreement with those reported.[19]

Yellow solid, m.p.: 119-121 °C; ^{1}H NMR (400 MHz, 300 K, $CDCl_3$): δ = 7.73 (d, *J* = 15.9 Hz, 2H), 7.70 – 7.61 (m, 4H), 7.27 (d, *J* = 8.0 Hz, 4H), 7.06 ppm (d, *J* = 16.0 Hz, 2H); ^{13}C NMR (100 MHz, 300 K, $CDCl_3$): δ = 188.2, 150.6 (q, *J* = 1.9 Hz), 141.8, 133.2, 129.8, 126.0, 121.2, 120.3 ppm (q, *J* = 258.2 Hz); ^{19}F NMR (376 MHz, 300 K, $CDCl_3$): δ = -57.8 ppm (s, 6F); IR (film): ṽ = 1651, 1580, 1508, 1263, 1214, 1190, 1164, 1109 cm^{-1}; HRMS (ESI): *m/z* calculated for $C_{19}H_{13}O_3F_6$ $[M+H]^+$: 403.0763, found: 403.0760.

(1*E*,4*E*)-1,5-Bis(2-methoxyphenyl)penta-1,4-dien-3-one (14)

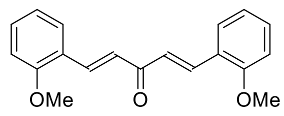

According to General Procedure A, diene **14** (2.29 g, 78%) was obtained from 2-methoxybenzaldehyde (2.72 g, 20 mmol) and acetone (0.58 g, 10 mmol), following column chromatography [50 g Sfär Silica D; 120 mL/min, 100% cyclohexane (2 CV), followed by a linear gradient (12 CV): 0%→20% acetone in cyclohexane]. The analytical data for **14** were in agreement with those reported.[20]

Yellow solid, m.p.: 125-128 °C; ^{1}H NMR (400 MHz, 300 K, $CDCl_3$): δ = 8.08 (d, *J* = 16.1 Hz, 2H), 7.62 (dd, *J* = 7.7, 1.6 Hz, 2H), 7.36 (ddd, *J* = 8.3, 7.4, 1.7 Hz, 2H), 7.18 (d, *J* = 16.1 Hz, 2H), 6.98 (t, *J* = 7.5 Hz, 2H), 6.92 (d, *J* = 7.9 Hz, 2H), 3.90 ppm (s, 6H); ^{13}C NMR (100 MHz, 300 K, $CDCl_3$): δ = 189.9, 158.5, 138.1, 131.5, 128.6, 126.1, 123.9, 120.6, 111.1, 55.4 ppm; IR (film): ṽ = 2838, 1666, 1647, 1612, 1598, 1573,

1487, 1464, 1437, 1336, 1296, 1274, 1246, 1184, 1163, 1106, 1049, 1026 cm^{-1}; HRMS (ESI): *m/z* calculated for $C_{19}H_{19}O_3$ $[M+H]^+$: 295.1329, found: 295.1330.

(1*E*,4*E*)-1,5-Bis(2-(trifluoromethoxy)phenyl)penta-1,4-dien-3-one (15)

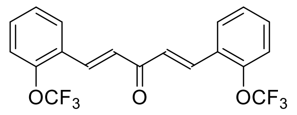

According to General Procedure A, diene **15** (2.53 g, 63%) was obtained from 2-(trifluoromethoxy)benzaldehyde (3.80 g, 20 mmol) and acetone (0.58 g, 10 mmol), following column chromatography [100 g Sfär Silica D; 120 mL/min, 100% cyclohexane (2 CV), followed by a linear gradient (12 CV): 0%→10% acetone in cyclohexane].

Yellow solid, m.p.: 75-78 °C; ^1H NMR (400 MHz, 300 K, $CDCl_3$): δ = 7.94 (d, *J* = 16.1 Hz, 2H), 7.74 (dd, *J* = 7.8, 1.7 Hz, 2H), 7.46 – 7.42 (m, 2H), 7.37 – 7.30 (m, 4H), 7.13 ppm (d, *J* = 16.1 Hz, 2H); ^{13}C NMR (100 MHz, 300 K, $CDCl_3$): δ = 188.5, 147.9, 136.3, 131.5, 128.4, 128.0, 127.8, 127.1, 121.3, 120.5 ppm (q, *J* = 258.6 Hz); ^{19}F NMR (376 MHz, 300 K, $CDCl_3$): δ = -57.3 ppm (s, 6F); IR (film) ṽ = 1660, 1623, 1603, 1487, 1457, 1334, 1248, 1210, 1170, 1098 cm^{-1}; HRMS (ESI): *m/z* calculated for $C_{19}H_{13}O_3F_6$ $[M+H]^+$: 403.0763, found: 403.0754.

(1*E*,4*E*)-1,5-Bis(3,5-dimethylphenyl)penta-1,4-dien-3-oneone (16)

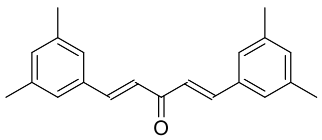

According to General Procedure A, diene **16** (0.64 g, 22%) was obtained from 3,5-dimethylbenzaldehyde (2.68 g, 20 mmol) and acetone (0.58 g, 10 mmol), following column chromatography [25 g Sfär Silica D; 80 mL/min, 100% cyclohexane (2 CV), followed by a linear gradient (12 CV): 0%→15% acetone in cyclohexane].

Yellow solid, m.p.: 109-111 °C; ^1H NMR (400 MHz, 300 K, $CDCl_3$): δ = 7.71 (d, *J* = 15.9 Hz, 2H), 7.27 – 7.25 (m, 4H), 7.13 – 7.05 (m, 4H), 2.38 ppm (s, 12H); ^{13}C NMR (100 MHz, 300 K, $CDCl_3$): δ = 188.9, 143.3, 138.3, 134.7, 132.2, 126.2, 125.1, 21.1 ppm; IR (film): ṽ = 2980, 2918, 1652, 1620, 1604, 1439, 1341, 1287, 1255, 1188, 1161, 1105 cm^{-1}; HRMS (ESI): *m/z* calculated for $C_{21}H_{23}O$ $[M+H]^+$: 291.1743, found: 291.1743.

(1*E*,4*E*)-1,5-Bis(2,6-dimethylphenyl)penta-1,4-dien-3-oneone (17)

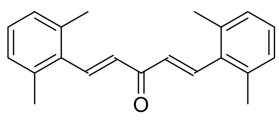

According to General Procedure A, diene **17** (0.20 g, 7%) was obtained from 2,6-dimethylbenzaldehyde (2.68 g, 20 mmol), and acetone (0.58 g, 10 mmol), following recrystallisation from *n*-pentane.

Yellow solid, m.p.: 150-153 °C; ^{1}H NMR (400 MHz, 300 K, $CDCl_3$): δ = 7.92 (d, *J* = 16.3 Hz, 2H), 7.19 – 7.09 (m, 6H), 6.74 (d, *J* = 16.3 Hz, 2H), 2.43 ppm (s, 12H); ^{13}C NMR (100 MHz, 300 K, $CDCl_3$): δ = 189.0, 142.1, 136.8, 134.4, 131.1, 128.4, 128.3, 21.1 ppm; IR (film): ṽ = 2981, 1650, 1625, 1591, 1467, 1446, 1382, 1355, 1291, 1192, 1165, 1004 cm^{-1}; HRMS (ESI): *m/z* calculated for $C_{21}H_{23}O$ $[M+H]^+$: 291.1743, found: 291.1743.

2,7-Di((*E*)-benzylidene)cycloheptan-1-one (18)

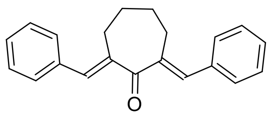

According to General Procedure A, diene **18** (0.78 g, 27%) was obtained from benzaldehyde (2.12 g, 20 mmol) and cycloheptanone (1.12 g, 10 mmol) following column chromatography [25 g Sfär Silica D; 80 mL/min, 100% cyclohexane (2 CV), followed by a linear gradient (12 CV): 0%→10% acetone in cyclohexane]. The analytical data for **18** were in agreement with those reported.[21, 22]

Yellow solid, m.p.: 104-108 °C; ^{1}H NMR (400 MHz, 300 K, $CDCl_3$): δ = 7.53 – 7.33 (m, 12H), 2.78 – 2.70 (m, 4H), 2.04 – 1.99 ppm (m, 4H); ^{13}C NMR (100 MHz, 300 K, $CDCl_3$): δ = 199.4, 141.7, 135.9, 135.6, 129.4, 128.4, 128.1, 28.8, 28.0 ppm; IR (film): ṽ = 2927, 1667, 1623, 1604, 1492, 1455, 1446, 1306, 1291, 1228, 1190, 1144, 1021 cm^{-1}; HRMS (ESI): *m/z* calculated for $C_{21}H_{21}O$ $[M+H]^+$: 289.1587, found: 289.1586.

(*trans*-3-Phenyloxiran-2-yl)(*trans*-3-phenyloxiran-2-yl)methanone (1)

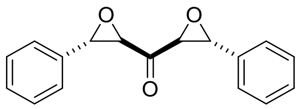

According to General Procedure B, DEK **1** (180 mg, 35%) was obtained as a single *trans*,*trans*-diastereomer from (1*E*,4*E*)-1,5-diphenylpenta-1,4-dien-3-one[16] (0.47 g, 2 mmol) following recrystallisation from ethanol. The stereochemistry of **1** was assigned as *trans*,*trans* by X-ray diffraction analysis of a single crystal obtained after recrystallisation (Table S4; CCDC 2262059). Note that the analysis of the crude reaction mixture using ^{1}H NMR analysis indicated that two diastereomers are formed during the reaction, one of which was assigned as the *trans*,*trans*-diastereomer; the other diastereomer formed is likely the *cis*,*cis*-diastereomer (*cis*,*trans*-diastereomeric mixtures should manifest in an additional set of signals for the epoxide protons and were not detected using ^{1}H NMR analysis). Note that both the *trans*,*trans*-diastereomer and the *cis*,*cis*-diastereomer are *meso*-compounds. The analytical data for **1** were in agreement with those reported.[23]

White crystals, m.p.: 119-120 °C. ^{1}H NMR (400 MHz, 300 K, $CDCl_3$): δ = 7.40 – 7.37 (m, 6H), 7.32 – 7.29 (m, 4H), 4.10 (d, *J* = 1.8 Hz, 2H), 3.81 ppm (d, *J* = 1.8 Hz, 2H); ^{13}C NMR (100 MHz, 300 K, $CDCl_3$): δ = 199.2, 134.6, 129.3, 128.8, 125.8, 60.9, 59.0 ppm; IR (film): ṽ = 3062, 1721, 1458, 1428, 1410, 1117, 1084 cm^{-1}; HRMS (ESI): *m/z* calculated for $C_{17}H_{15}O_3$ $[M+H]^+$: 267.1016, found: 267.1015.

Purification of the mother liquor obtained after recrystallization using column chromatography (10 g Sfär Silica D; 40 mL/min, 100% cyclohexane (2 CV), followed by a linear gradient (12 CV): 0%→20% ethyl acetate in cyclohexane) afforded a 3:1 mixture of *cis*,*cis*:*trans*,*trans*-**1** (37 mg, 7%) as an orange oil. Note that *cis*,*cis*-enriched **1** appears to be an oil and not a solid like *trans*,*trans* **1**; as yet *cis*,*cis*-enriched **1** has not been further purified by recrystallisation or trituration. By implication, subsequently acquired diastereomerically pure αβ,α′β′-diepoxides obtained by crystallisation were *tentatively* assigned as the *trans*,*trans*-diastereomers if they were obtained as pure solids. ^{1}H and ^{13}C NMR signals observed for *cis*,*cis*-enriched **1**: ^{1}H NMR (400 MHz, 300 K, $CDCl_3$): δ = 4.20 (d, *J* = 1.7 Hz, 2H), 3.74 ppm (d, *J* = 1.7 Hz, 2H); ^{13}C NMR(100 MHz, 300 K, $CDCl_3$): δ = 199.2, 134.5, 129.2, 128.7, 125.8, 60.3, 58.8 ppm.

(*trans*-3-(4-(Trifluoromethoxy)phenyl)oxiran-2-yl)(*trans* 3-(4-(trifluoromethoxy)phenyl)oxiran-2-yl)methanone (4)

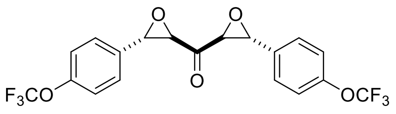

According to General Procedure B, DEK **4** (95 mg, 11%) was obtained as a single diastereomer from diene **13** (0.81 g, 2 mmol) following recrystallisation from ethanol. DEK **4** was *tentatively* assigned as the *trans*,*trans*-diastereomer, in part because it was obtained as a solid after recrystallization; *trans*,*trans*-**1** was a solid and its stereochemistry was assigned by crystallographic analysis, whereas *cis*,*cis*-**1b** manifested as an oil.

White crystals, m.p.: 95-97 °C; ^{1}H NMR (400 MHz, 300 K, $CDCl_3$): δ = 7.38 – 7.34 (m, 4H), 7.28 – 7.25 (m, 4H), 4.16 (d, *J* = 1.8 Hz, 2H), 3.79 ppm (d, *J* = 1.8 Hz, 2H); ^{13}C NMR (100 MHz, 300 K, $CDCl_3$): δ = 198.4, 149.9 (q, *J* = 1.9 Hz), 133.2, 127.3, 121.4, 120.6 (q, *J* = 257.8 Hz), 60.9, 58.1 ppm; ^{19}F NMR (376 MHz, 300 K, $CDCl_3$): δ = -57.9 ppm (s, 6F); IR (film): ṽ = 1718, 1514, 1260, 1210, 1162 cm^{-1}; HRMS (ESI): *m/z* calculated for $C_{19}H_{12}O_5F_6Na$ $[M+Na]^+$: 457.0481, found: 457.0481.

(3-(2-Methoxyphenyl)oxiran-2-yl)(3-(2-methoxyphenyl)oxiran-2-yl)methanone (5)

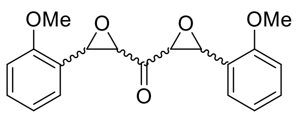

According to General Procedure B, DEK **5** (189 mg, 29%) was obtained from diene **14** (0.59 g, 2 mmol) as a 2:1 mixture of diastereomers (likely the *trans*,*trans*- and *cis*,*cis*-diastereomers) following column chromatography (10 g Sfär Silica D; 40 mL/min, 100% cyclohexane (2 CV), followed by a linear gradient (12 CV): 0%→20% ethyl acetate in cyclohexane).

Analytical data for the ~2:1 mixture of diastereomers: ^{1}H NMR (400 MHz, 300 K, $CDCl_3$): δ = 7.36 – 7.31 (m, 6H), 7.23 – 7.18 (m, 6H), 7.00 – 6.91 (m, 12H), 4.51 (d, *J* = 1.8 Hz, 2H), 4.48 (d, *J* = 1.9 Hz, 4H), 3.90 (s, 12H), 3.88 (s, 6H), 3.74 (d, *J* = 1.8 Hz, 4H), 3.67 ppm (d, *J* = 1.8 Hz, 2H); ^{13}C NMR (100 MHz, 300 K,

$CDCl_3$): δ = 200.1, 200.0, 158.3, 158.0, 129.8(4), 129.8, 125.3, 125.2, 123.6, 123.2, 120.8, 120.6, 110.4, 110.2, 60.3, 59.8, 55.4, 55.3(2), 55.3, 55.0 ppm; HRMS (ESI): *m/z* calculated for $C_{19}H_{19}O_5$ $[M+H]^+$: 327.1227, found: 327.1227.

(*trans*-3-(2-(Trifluoromethoxy)phenyl)oxiran-2-yl)(*trans*-3-(2-(trifluoromethoxy)phenyl)oxiran-2-yl)methanone (6)

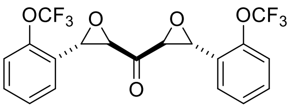

According to General Procedure B, DEK **6** (191 mg, 22%) was obtained as a single diastereomer from diene **15** (0.81 g, 2 mmol) following recrystallisation from ethanol. DEK **6** was *tentatively* assigned as the *trans*,*trans*-diastereomer, in part because it was obtained as a solid after recrystallization (as described above).

White crystals, m.p.: 136-139 °C. ^1H NMR (400 MHz, 300 K, $CDCl_3$): δ = 7.43 – 7.27 (m, 8H), 4.40 (d, *J* = 1.8 Hz, 2H), 3.71 ppm (d, *J* = 1.8 Hz, 2H); ^{13}C NMR (100 MHz, 300 K, $CDCl_3$): δ = 198.0, 147.8, 130.2, 128.0, 127.5, 125.8, 121.1, 120.5 (q, *J* = 257.6 Hz), 60.1, 54.0 ppm; ^{19}F NMR (376 MHz, 300 K, $CDCl_3$): δ = -57.9 ppm (s, 6F); IR (film): ṽ = 1720, 1274, 1252, 1207, 1183, 1165, 1100, 1077 cm^{-1}; HRMS (ESI): *m/z* calculated for $C_{19}H_{12}O_5F_6Na$ $[M+Na]^+$: 457.0481, found: 457.0479.

(*trans*-3-(3,5-Dimethylphenyl)oxiran-2-yl)(*trans*-3-(3,5-dimethylphenyl)oxiran-2-yl)methanone (7)

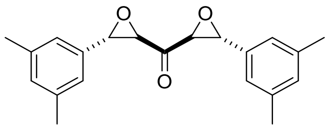

According to General Procedure B, DEK **7** (100 mg, 16%) was obtained as a single diastereomer from diene **16** (0.59 g, 2 mmol) following recrystallisation from ethanol. DEK **7** was *tentatively* assigned as the *trans*,*trans*-diastereomer, in part because it was obtained as a solid after recrystallization (as described above).

White crystals, m.p.: 124-128 °C. ^1H NMR (400 MHz, 300 K, $CDCl_3$): δ = 6.99 (s, 2H), 6.92 (s, 4H), 4.09 (d, *J* = 1.7 Hz, 2H), 3.69 (d, *J* = 1.7 Hz, 2H), 2.32 ppm (s, 12H); ^{13}C NMR (100 MHz, 300 K, $CDCl_3$): δ = 199.5, 138.5, 134.4, 130.9, 123.5, 60.2, 58.9, 21.2 ppm; IR (film): ṽ = 2920, 1716, 1609, 1470, 1408, 1225, 1189, 1071, 1038 cm^{-1}; HRMS (ESI): *m/z* calculated for $C_{21}H_{22}O_3Na$ $[M+Na]^+$: 345.1461, found: 345.1463.

(3-(2,6-Dimethylphenyl)oxiran-2-yl)(3-(2,6-dimethylphenyl)oxiran-2-yl)methanone (8)

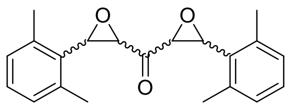

According to General Procedure B, DEK **8** (38 mg, 24%) was obtained from diene **17** (145 mg, 0.5 mmol) as a 1.2:1 mixture of diastereomers (likely the *trans*,*trans*- and *cis*,*cis*-diastereomers) following column chromatography (5

g Sfär Silica D; 18 mL/min, 100% cyclohexane (2 CV), followed by a linear gradient (12 CV): 0%→10% ethyl acetate in cyclohexane).

Analytical data of the ~1.2:1 mixture of diastereomers: ^{1}H NMR (400 MHz, 300 K, $CDCl_3$): δ = 7.17 (t, *J* = 7.6 Hz, 4H), 7.03 (d, *J* = 7.6 Hz, 8H), 4.32 (d, *J* = 2.0 Hz, 2H), 4.24 (d, *J* = 1.9 Hz, 2H), 3.78 (d, *J* = 2.1 Hz, 2H), 3.68 (d, *J* = 2.0 Hz, 2H), 2.43 (s, 12H), 2.42 ppm (s, 12H); ^{13}C NMR (100 MHz, 300 K, $CDCl_3$): δ = 201.9, 201.8(5), 137.2, 136.9, 131.5, 131.4(7), 128.5, 128.3, 128.2, 58.6, 58.3, 57.9, 57.8(6), 19.9, 19.7 ppm; HRMS (ESI): *m/z* calculated for $C_{21}H_{23}O_3$ [M+H]$^+$: 323.1642, found: 323.1642.

(*trans*)-2,7-Diphenyl-1,6-dioxadispiro[2.1.2^5.2^3]nonan-4-one (9)

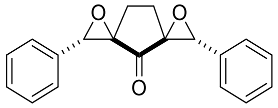

According to General Procedure B, DEK **9** (300 mg, 52%) was obtained as a single diastereomer from 2,5-di((*E*)-benzylidene)cyclopentan-1-one[16] (0.52 g, 2 mmol) without further purification. DEK **9** was *tentatively* assigned as the *trans*,*trans*-diastereomer, in part because it was obtained as a solid.

White solid, m.p.: 115-118 °C. ^{1}H NMR (400 MHz, 300 K, $CDCl_3$): δ = 7.35 – 7.27 (m, 6H), 7.21 – 7.18 (m, 4H), 4.34 (s, 2H), 1.97 – 1.87 (m, 2H), 1.79 – 1.70 ppm (m, 2H); ^{13}C NMR (100 MHz, 300 K, $CDCl_3$): δ = 208.6, 133.4, 128.8, 128.5, 126.6, 66.1, 65.2, 19.9 ppm; IR (film): ṽ = 1762, 1410 cm^{-1}; HRMS (ESI): *m/z* calculated for $C_{19}H_{16}O_3Na$ [M+Na]$^+$: 315.0992, found: 315.0991.

(*trans*)-2,7-Diphenyl-1,6-dioxadispiro[2.1.2^5.2^3]decan-4-one (10)

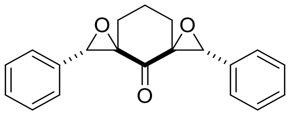

According to General Procedure B, DEK **10** (270 mg, 44%) was obtained as a single diastereomer from 2,6-di((*E*)-benzylidene)cyclohexan-1-one[16] (0.55 g, 2 mmol) without further purification. DEK **10** was *tentatively* assigned as the *trans*,*trans*-diastereomer, in part because it was obtained as a solid.

White solid, m.p.: >230 °C (decomposition). ^{1}H NMR (400 MHz, 300 K, $CDCl_3$): δ = 7.44 – 7.30 (m, 10H), 4.15 (s, 2H), 2.15 – 2.07 (m, 2H), 1.65 – 1.58 (m, 3H), 1.25 – 1.11 ppm (m, 1H); ^{13}C NMR (100 MHz, 300 K, $CDCl_3$): δ = 203.1, 133.1, 128.6, 128.5, 126.6, 66.1, 65.5, 26.0, 19.4 ppm; IR (film): ṽ = 1716, 1453, 1407 cm^{-1}; HRMS (ESI): *m/z* calculated for $C_{20}H_{18}O_3Na$ [M+Na]$^+$: 329.1148, found: 329.1148.

(*trans*)-2,7-Diphenyl-1,6-dioxadispiro[2.1.2^5.2^3]undecan-4-one (11)

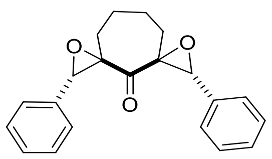

According to General Procedure B, DEK **11** (260 mg, 41%) was obtained as a single diastereomer from diene **18** (0.58 g, 2 mmol) without further purification. DEK **11** was *tentatively* assigned as the *trans*,*trans*-diastereomer, in part because it was obtained as a solid.

White solid, m.p.: 147-150 °C. ^{1}H NMR (400 MHz, 300 K, $CDCl_3$): δ = 7.40 – 7.32 (m, 10H), 4.23 (s, 2H), 1.90 – 1.79 (m, 4H), 1.49 – 1.46 (m, 2H), 1.35 – 1.31 ppm (m, 2H); ^{13}C NMR (100 MHz, 300 K, $CDCl_3$): δ = 203.6, 133.6, 128.4, 128.3, 126.6, 67.7, 63.9, 26.9, 24.4 ppm; IR (film): ṽ = 1718, 1454 cm^{-1}; HRMS (ESI): *m/z* calculated for $C_{21}H_{20}O_3Na$ $[M+Na]^+$: 343.1305, found: 345.1306.

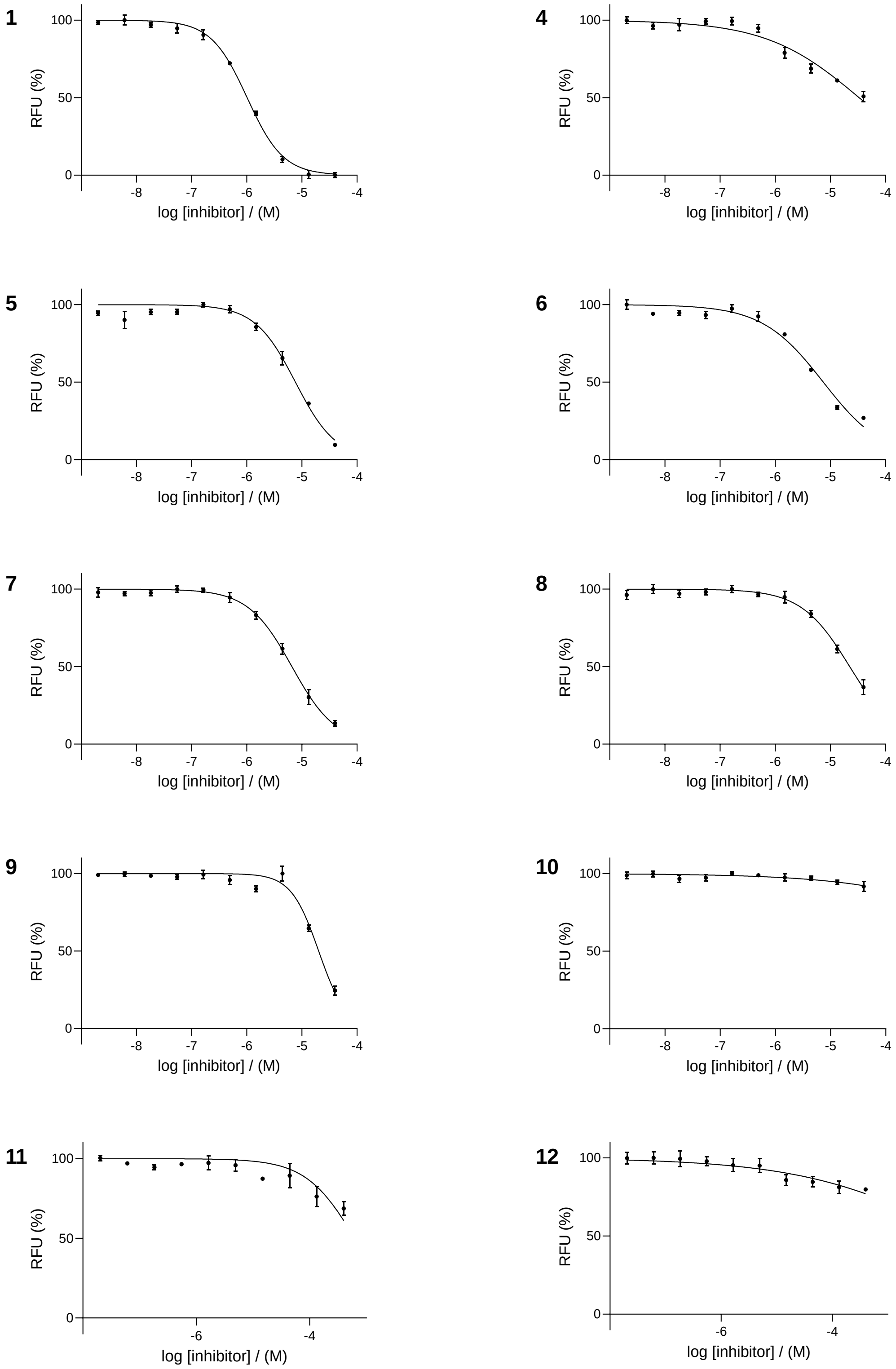

Figure S1. Dose-response curves for the DEKs 1 and 4-11 and mono-epoxide ketone 12 with Ldt_{Mt2}. Inhibition assays were carried out using 100 nM Ldt_{Mt2} and 25 µM Probe 1 with 10 min pre-incubation at room temperature in 50 mM HEPES, pH 7.2 with 0.01% (*v/v*) Triton X-100. Error bars represent standard deviation (n=4). Average pIC_{50} values and compound structures are given in Table S3.

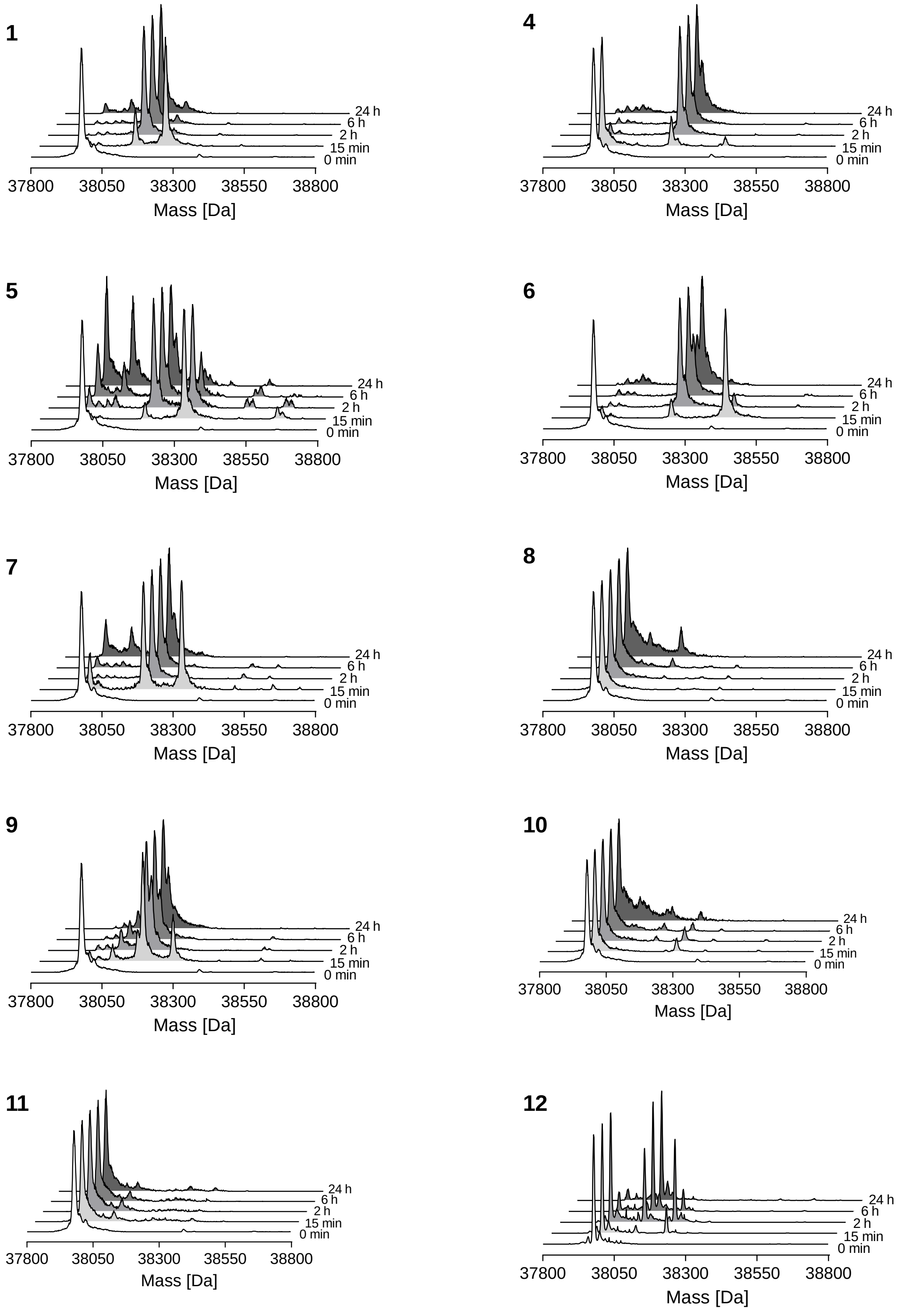

Figure S2. Protein observed SPE-MS analysis for the reaction of Ldt_{Mt2} with DEKs 1 and 4-11 and mono-epoxide ketone 12. 1 µM Ldt_{Mt2} was incubated with the inhibitors (20 µM for **1** and **4** – **11**, 100 µM for **12**) at rt in 50 mM Tris, pH 7.5. Samples were analysed after the indicated times. Deconvoluted spectra, obtained using the maximum entropy algorithm in the MassHunter Workstation Qualitative Analysis B.07.00 program (Agilent), are shown. Mass shifts and assignments are given in Table S1.

Table S1. Calculated and observed masses (Da) and mass shifts (Da) for protein-observed SPE-MS experiments with Ldt$_{Mt2}$ and inhibitors 1 and 4-12, and their assignments. Mass shifts are relative to unmodified Ldt$_{Mt2}$. The observed mass of the most abundant adduct at 24h is in blue. *Calculated mass for the fragmented adduct **3** (as shown in Figure 2). **Calculated mass for the unfragmented adduct **2** (as shown in Figure 2). Deconvoluted SPE-MS spectra are shown in Figure S2.

Compound	Calculated mass (Da)	Observed mass (Da)	Area (%) 15 min	Area (%) 24 h	Assignment
**1**	39139 (+161)*<br>38245 (+267)**	37944 (-34)	0.0	5.0	Dha
		37978 (+0)	2.0	0.0	Unmodified
		38034 (+56)	0.0	5.9	Unassigned fragment[1]
		38138 (+160)	23.3	82.0	Retro-aldol fragment (**3**)
		38245 (+267)	74.7	7.2	Unfragmented (**2**)
**4**	38223 (+245)*<br>38413 (+435)**	37944 (-34)	0.0	2.4	Dha
		37978 (+0)	76.5	0.0	Unmodified
		38221 (+244)	18.1	97.6	Retro-aldol fragment (**3**)
		38412 (+434)	5.41	0.0	Unfragmented (**2**)
**5**	38169 (+191)*<br>38305 (+327)**	37944 (-34)	0.0	30.4	Dha
		37978 (+0)	2.5	0.0	Unmodified
		38035 (+57)	0.0	25.8	Unassigned fragment[1]
		38169 (+191)	9.0	39.6	Retro-aldol fragment (**3**)
		38287 (+309)	0.0	4.22	N.D.
		38306 (+328)	77.9	0.0	Unfragmented (**2**)
**6**	38223 (+245)*<br>38413 (+435)**	37978 (+0)	7.2	2.6	Unmodified
		38032 (+56 Da)	0.0	4.2	Fragment II[1]
		38221 (+243)	11.6	93.2	Retro-aldol fragment (**3**)
		38413 (+435)	81.2	0.0	Unfragmented (**2**)
**7**	38167 (+189)*<br>38210 (+232)**	37944 (-34)	0.0	16.2	Dha
		37978 (+0)	11.5	0.0	Unmodified
		38034 (+56)	0.0	13.5	Unassigned fragment[1]
		38166 (+188)	43.7	70.3	Retro-aldol fragment (**3**)
		38301 (+232)	44.8	0.0	Unfragmented (**2**)
**8**	38167 (+189)*<br>38210 (+232)**	37978 (+0)	100.0	68.8	Unmodified
		38058 (+80)	0.0	14.9	N.D.
		38166 (+188)	0.0	16.3	Retro-aldol fragment (**3**)
**9**	38165 (+187)*<br>38271 (+293)**	37978 (+0)	3.3	0.0	Unmodified
		38058 (+80)	6.9	9.1	N.D.
		38163 (+185)[2]	68.8	90.9	Retro-aldol fragment (**3**)
		38271 (+293)	21.0	0.0	Unfragmented (**2**)
**10**	38179 (+201)*<br>38285 (+307)**	37978 (+0)	100.0	90.2	Unmodified
		38285 (+307)	0.0	9.8	Unfragmented (**2**)
**11**	38193 (+215)*<br>38299 (+321)**	37978 (+0)	100.0	100.0	Unmodified
**12**	38097 (+119)*<br>38202 (+224)**	37978 (+0)	91.4%	12.8%	Unmodified
		38097 (+119)	0.0%	87.2%	Retro-aldol fragment (**3**)
		38202 (+224)	8.6%	0.0%	Unfragmented (**2**)

[1] See Figure S6 for possible structures of the unassigned fragment.

[2] The 38163 (+185) Da adduct was observed to shift to a mass of 38146 (+168) Da over the course of 24h, suggesting further fragmentation.

Table S2. Data collection and refinement statistics for the crystal structure of Ldt_{Mt2} reacted with 1.

**Datasets**	**Ldt_{Mt2} – 1 (PDB: 8BK3)**
**Data Collection**	
Beamline (Wavelength, Å)	DLS I03 (0.9763)
Detector	Eiger2 XE 16M
Data Processing	Xia2 dials
Space group	$P\ 1\ 2_1\ 1$
Cell dimensions	
a,b,c (Å)	60.93, 95.06, 75.53
α, β, γ (°)	90.00, 92.60, 90.00
No. of molecules/ASU	2
No. reflections	46835 (4654)*
Resolution (Å)	75.46-2.15 (2.23-2.15)*
R_{merge} (I)	0.167 (1.351)*
I/σI	10.2 (1.1)*
CC-1/2	0.97 (0.7)*
Completeness (%)	100.0 (99.5)*
Multiplicity	7.0 (7.0)*
Wilson B value (Å^2)	28.02
**Refinement**	PHENIX
R_{work}/R_{free}	0.2176/0.2487
No. atoms	6129
- Enzyme	5340
- Ligand	46
- Water	743
Average B-factors	36.62
- Enzyme	36.27
- Ligand	40.92
- Water	38.86
RMS[$] deviations	
- Bond lengths (Å)	0.005
- Bond angles (°)	0.58

[#] ASU = asymmetric unit.

[$] RMS = root mean square.

*Highest resolution shell in parentheses.

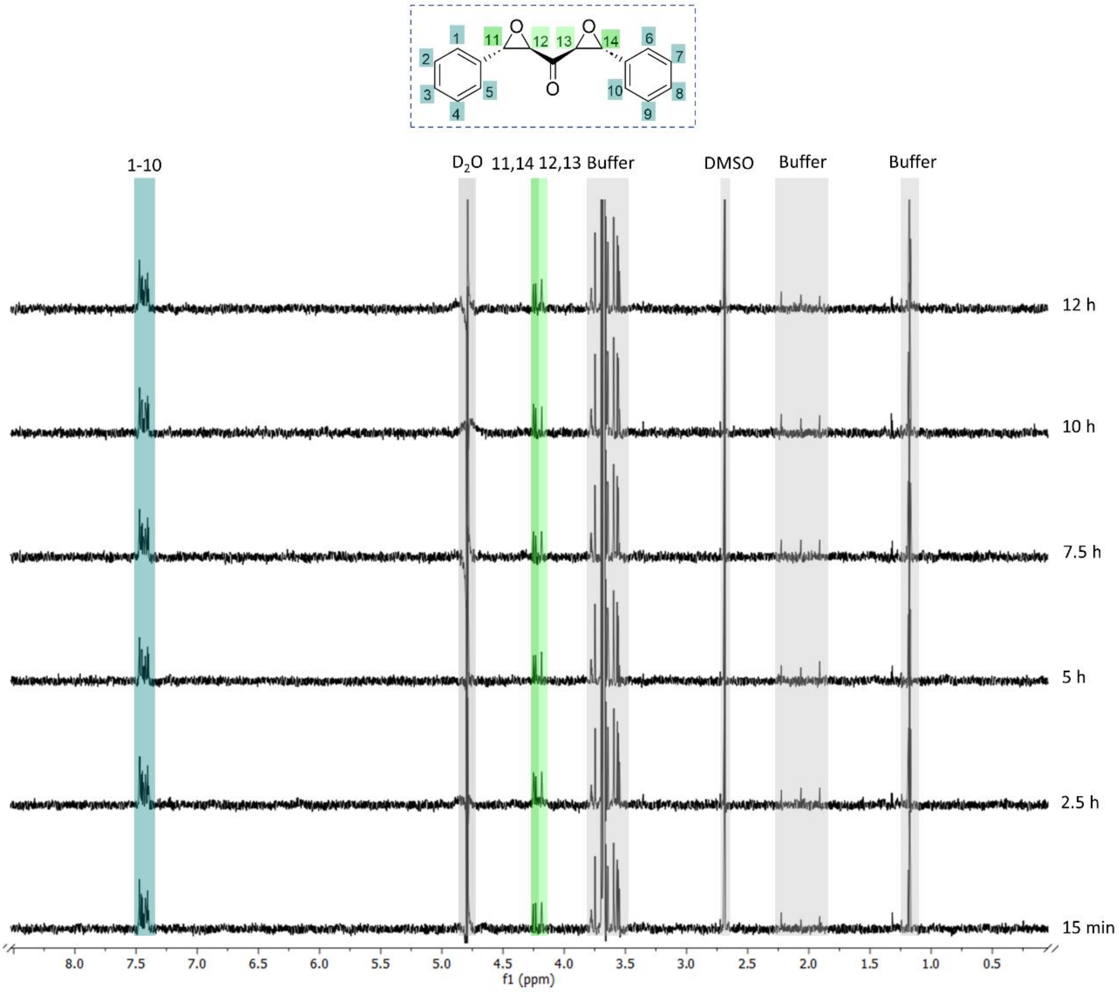

Figure S3. Stability of DEK 1 in aqueous solution. A solution of DEK **1** (250 µM) in 50 mM tris-d11, pH 7.5, 10% D_2O, was analysed by ^{1}H-NMR (950 MHz) for up to 12 h. No changes in peaks corresponding to **1** (in blue and green) in spectra were observed. Buffer and solvent peaks are in grey.

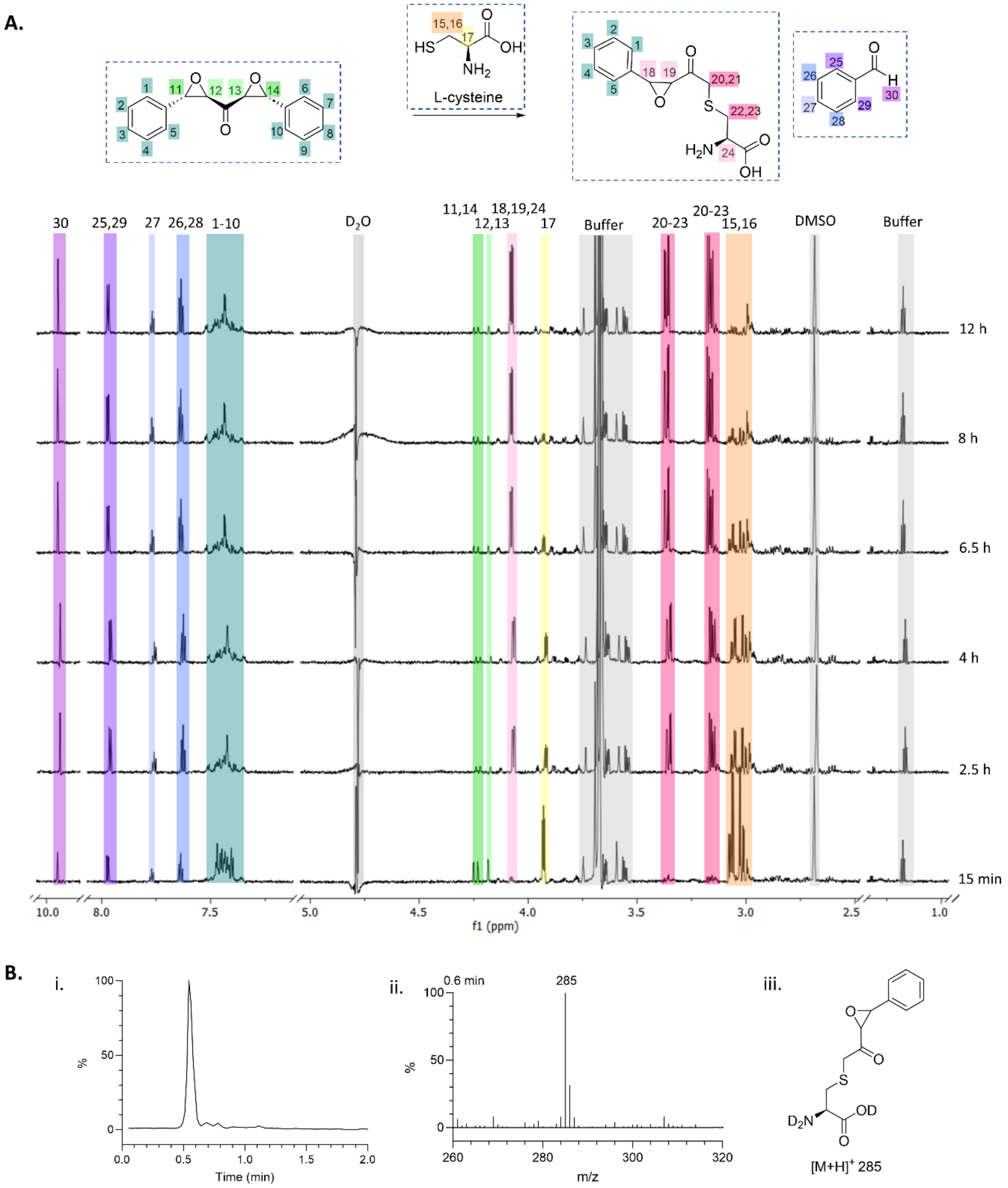

Figure S4. Reactivity of 1 with L-cysteine. A solution of **1** (250 µM) was incubated with L-cysteine (250 µM) in 50 mM tris-d11, pH 7.5, 10% D_2O. **A**. The reaction was analysed by ^{1}H-NMR (950 MHz) for up to 12 h. **B**. The reaction was analysed by LCMS, 16 h after initiation of the reaction.

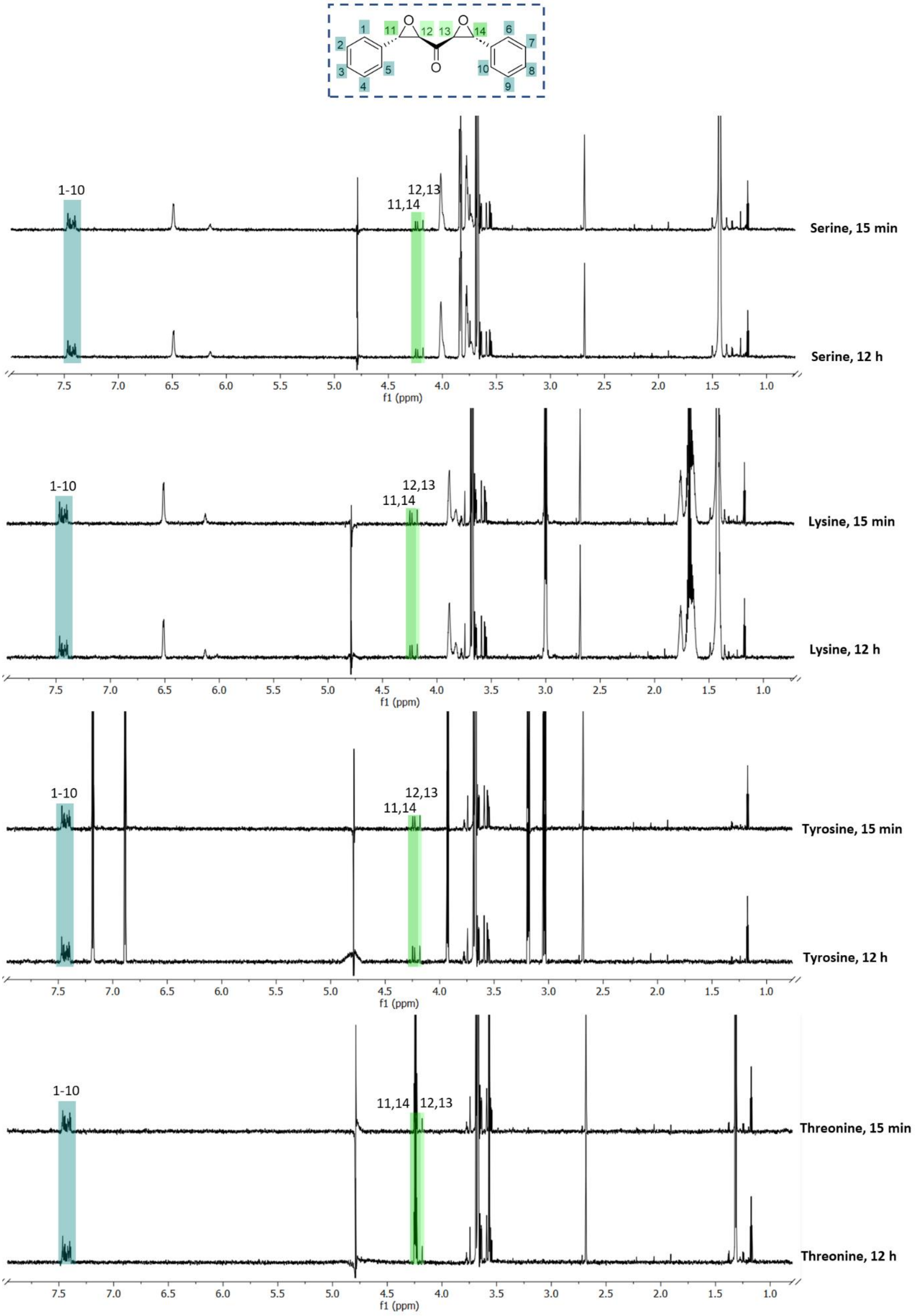

Figure S5. Reactivity of 1 with serine, lysine, tyrosine, threonine, arginine, and histidine. [Continues]

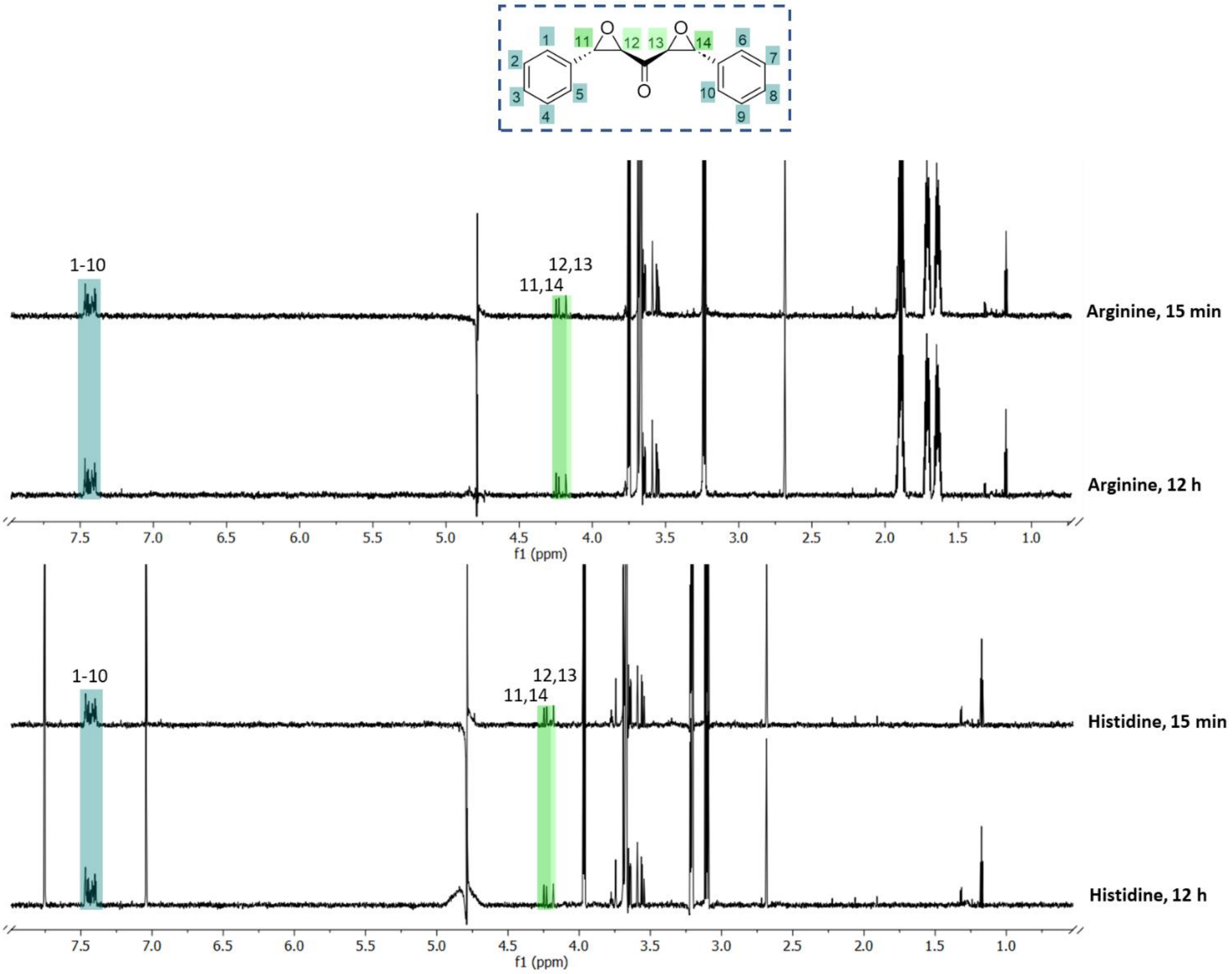

Figure S5. Reactivity of 1 with serine, lysine, tyrosine, threonine, arginine, and histidine. A solution of **1** (250 µM) was incubated with Boc-Ser-OH, Boc-Lys-OH, Tyr, Thr, Arg, or His, (250 µM) in 50 mM tris-d11, pH 7.5, 10% D_2O. The reaction was analysed by ^{1}H-NMR (950 MHz) up to 12 h. DEK **1** apparently did not react with the tested amino acids under the tested conditions.

Table S3. Synthesis and inhibitory characterisation of DEKs 1 and 4-12 with the nucleophilic cysteine enzymes Ldt_{Mt2} and SARS-CoV-2 M^{pro} and with the nucleophilic serine enzyme BlaC.

	Structure	Synthetic yield (%)		pIC50		k_{inact}/K_I (M^{-1} s^{-1})	k_{chem}
		Step 1[a]	Step 2 (dr)[a,b]	Ldt_{Mt2}	M^{pro}	Ldt_{Mt2}	(M^{-1} s^{-1})
**1**	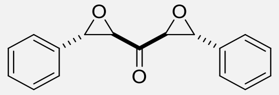	96	35 (1:0)	6.0 ± 0.03	4.6 ± 0.3	484.3 ± 28.4	<0.8
**4**	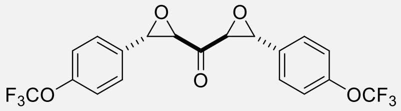	59	11 (1:0)	4.5 ± 0.1	<4.4	<10.0	<0.8
**5**	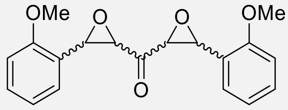	78	29 (2:1)	5.1 ± 0.05	<4.4	61.1 ± 2.78	<0.8
**6**	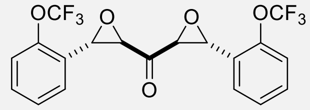	63	22 (1:0)	5.1 ± 0.06	<4.4	140.6 ± 17.1	<0.8
**7**	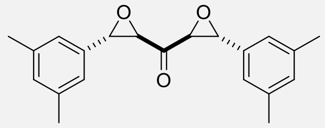	22	16 (1:0)	5.2 ± 0.03	<4.4	80.9 ± 6.74	1.7 ± 0.2
**8**	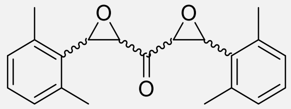	7	24 (1.2:1)	4.7 ± 0.04	<4.4	<10.0	1.1 ± 0.2
**9**	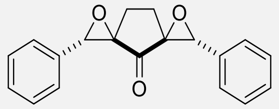	94	52 (1:0)	4.7 ± 0.04	5.9 ± 0.2	25.8 ± 1.41	<0.8
**10**	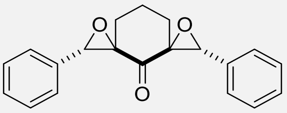	58	44 (1:0)	<4.4	<4.4	<10.0	<0.8
**11**	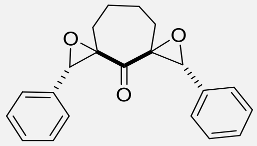	27	41 (1:0)	<4.4	<4.4	N.D.	N.D.
**12**	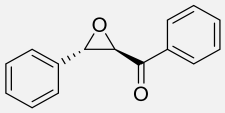	-	- (1:0)	<4.4	<4.4	N.D.	N.D.

[a] The 2-step synthesis involved the formation of the diene ketones (Step 1) followed by epoxidation (Step 2), to yield stereoisomeric mixtures (Figure 1D).

[b] Diastereomeric ratio (*trans*,*trans*:*cis*,*cis*) following purification, as determined by ^{1}H NMR analysis.

Table S4. Crystal data and structure refinement for 1.

**Datasets**	**1 (CCDC 2262059)**	
Empirical formula	C17 H14 O3	
Formula weight	266.30	
Temperature	150 K	
Wavelength	1.54184 Å	
Crystal system	Monoclinic	
Space group	C 2/c	
Cell dimensions	a = 28.7921(3) Å	α = 90°
	b = 5.34270(10) Å	β = 92.7637(11)°
	c = 8.59030(10) Å	γ = 90°
Volume	1319.89(3) $Å^3$	
Z	4	
Density (calculated)	1.340 Mg/m^3	
Absorption coefficient	0.742 mm^{-1}	
F(000)	560	
Crystal size	0.28 x 0.11 x 0.02 mm^3	
Theta range for data collection	3.073 to 75.928°	
Index ranges	-36<=h<=36, -6<=k<=6, -10<=l<=10	
Reflections collected	16701	
Independent reflections	1381 [R(int) = 0.024]	
Completeness to theta = 75.928°	99.9 %	
Absorption correction	Semi-empirical from equivalents	
Max. and min. transmission	0.99 and 0.71	
Refinement method	Full-matrix least-squares on F^2	
Data / restraints / parameters	1381 / 0 / 93	
Goodness-of-fit on F^2	0.9994	
Final R indices [I>2sigma(I)]	R1 = 0.0294, wR2 = 0.0777	
R indices (all data)	R1 = 0.0303, wR2 = 0.0786	
Extinction coefficient	12(2)	
Largest diff. peak and hole	0.25 and -0.13 e.$Å^{-3}$	

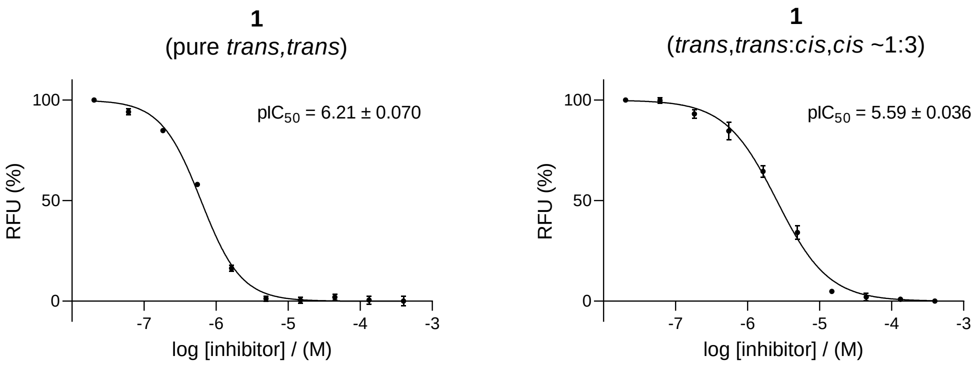

Figure S6. Dose-response curves for the pure *trans,trans* isomer of 1 and an isomeric mixture of 1 (*trans,trans*:*cis,cis* of ~1:3). Inhibition assays were carried out using 100 nM Ldt_{Mt2} and 25 µM Probe 1 with 15 min pre-incubation at rt in 50 mM HEPES, pH 7.2 with 0.01% (*v/v*) Triton X-100. Error bars represent the standard deviation (n=4). Note that pIC_{50} values are similar, but imply that the pure *trans,trans* isomer of **1** is the most active stereoisomer.

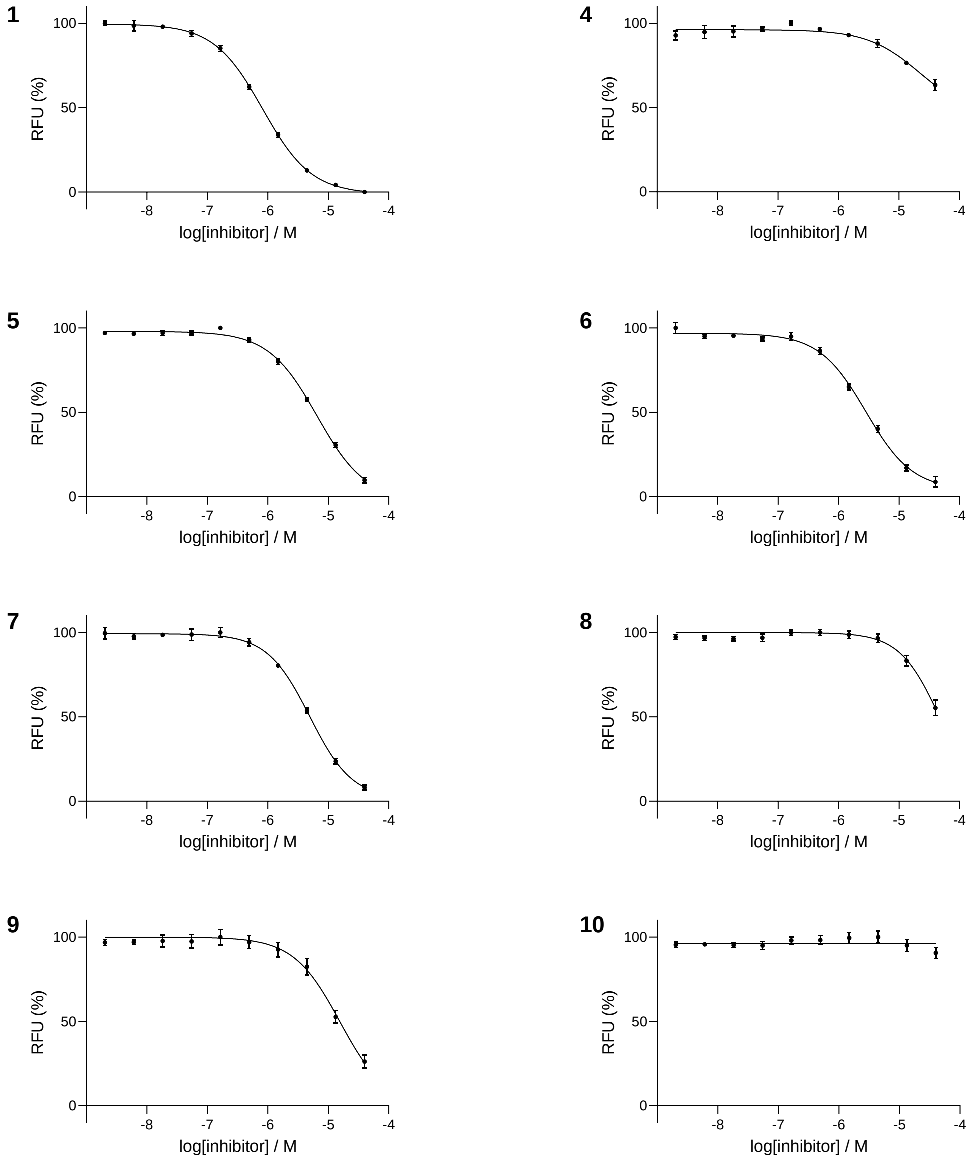

Figure S7. Determination of the second-order rate constant for irreversible inhibition (k_{inact}/K_I) of Ldt_{Mt2} by DEKs 1 and 4-10. Inhibition assays were carried out using 100 nM Ldt_{Mt2} and 10 µM Probe 1 with 3 h incubation at rt in 50 mM HEPES, pH 7.2 with 0.01% (*v/v*) Triton X-100. Error bars represent the standard deviation (n=4). Average $(k_{inact}/K_I)_{inhibitor}$ values and compound structures are given in Table S3.

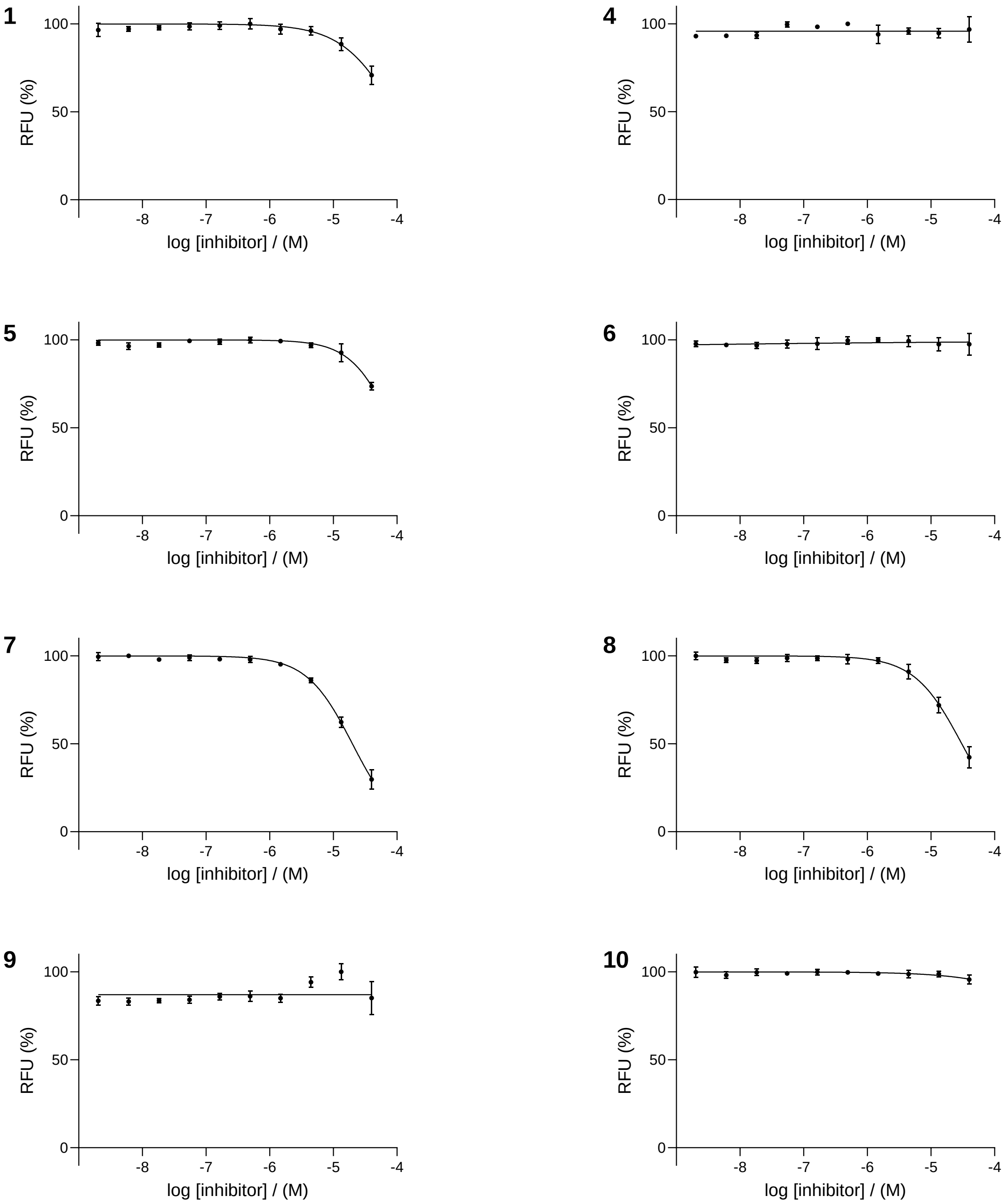

Figure S8. Determination of the intrinsic thiol reactivity rate constant (k_{chem}) for DEKs 1 and 4-10. Assays were carried out using 500 nM *L*-glutathione and 10 µM Probe 1 with 16 h incubation at room temperature in 50 mM HEPES, pH 7.2 with 0.01% (*v/v*) Triton X-100. Error bars represent the standard deviation (n=4). Average k_{chem} values and compound structures are given in Table S3.

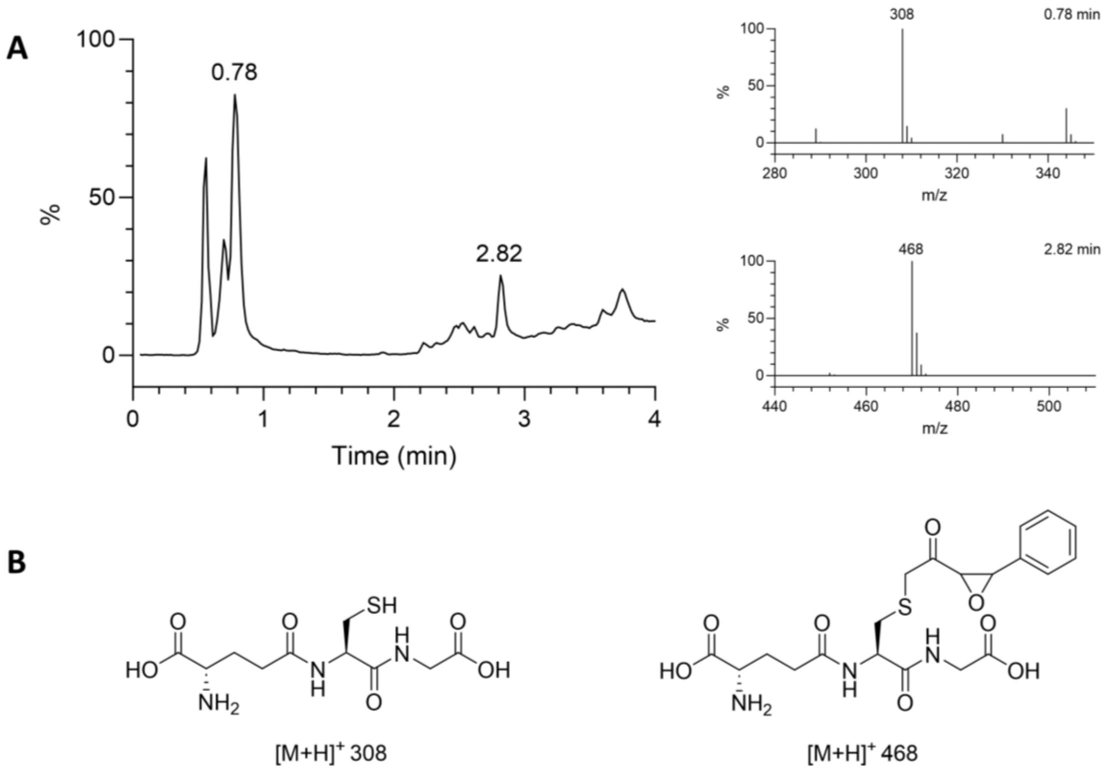

Figure S9. LCMS studies of the reaction between GSH and 1. A. A solution of **1** (250 µM) and GSH (250 µM) in 50 mM tris pH 7.5 was incubated for 16 h in the presence of TCEP (250 µM), then analysed by LCMS operating in the positive ion mode. **B**. The compounds eluting at 0.78 min and 2.82 min correspond to unreacted GSH and GSH reacted with **1** (apparently leading to the fragmented species analogous to **3**), respectively.

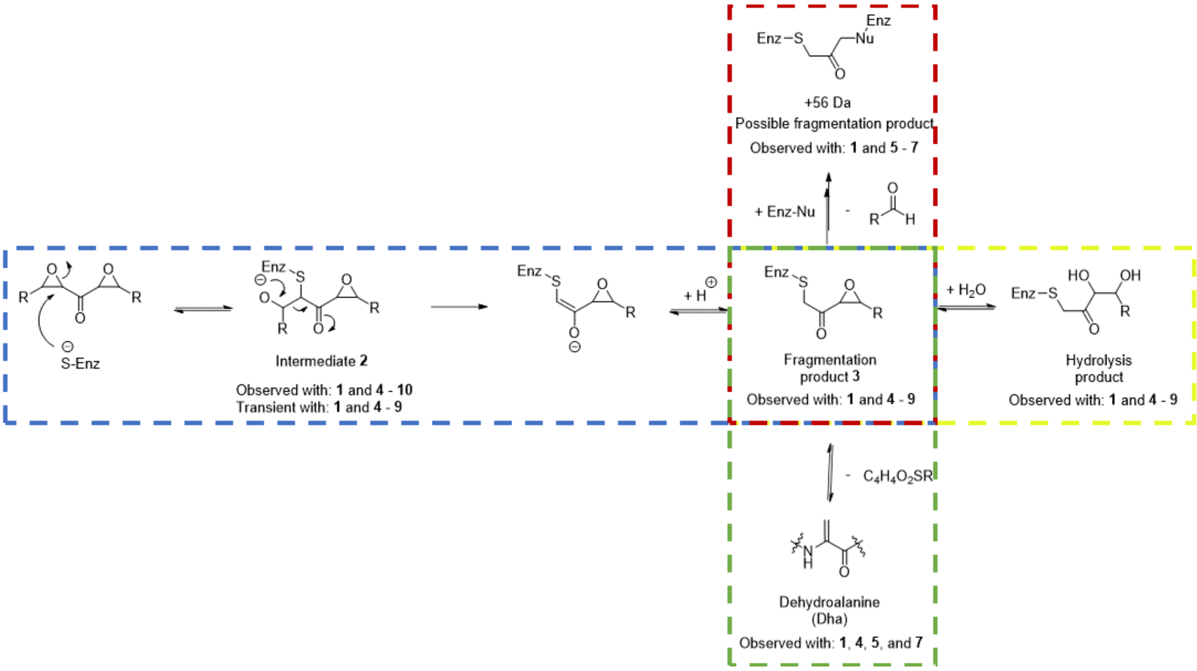

Figure S10. Summary of the reactions of DEKs with nucleophilic cysteine enzymes. The DEKs are proposed to inhibit nucleophilic cysteine enzymes via initial reaction of the nucleophilic cysteine with one of the epoxides, followed by retro-aldol reaction (blue outline). Initial reaction at the DEK carbonyl followed by rearrangement is also possible (Figure 2C). The fragmentation product **3** (Figure 2) obtained after retro-aldol reaction was sometimes (as indicated by compound numbers) observed (by MS analysis) to further react to give: (i) the corresponding hydrolysis product, likely through hydrolytic ring opening of its epoxide (yellow outline), or (ii) a dehydroalanine (Dha) residue (green outline). Another unassigned product corresponding to a mass shift of +57 Da compared to the unmodified enzyme was (sometimes) observed (red outline). Among other possibilities, the +57 Da fragmentation product(s) may arise through reaction with another nucleophilic residue in the active site, leading to a cross-linked adduct.

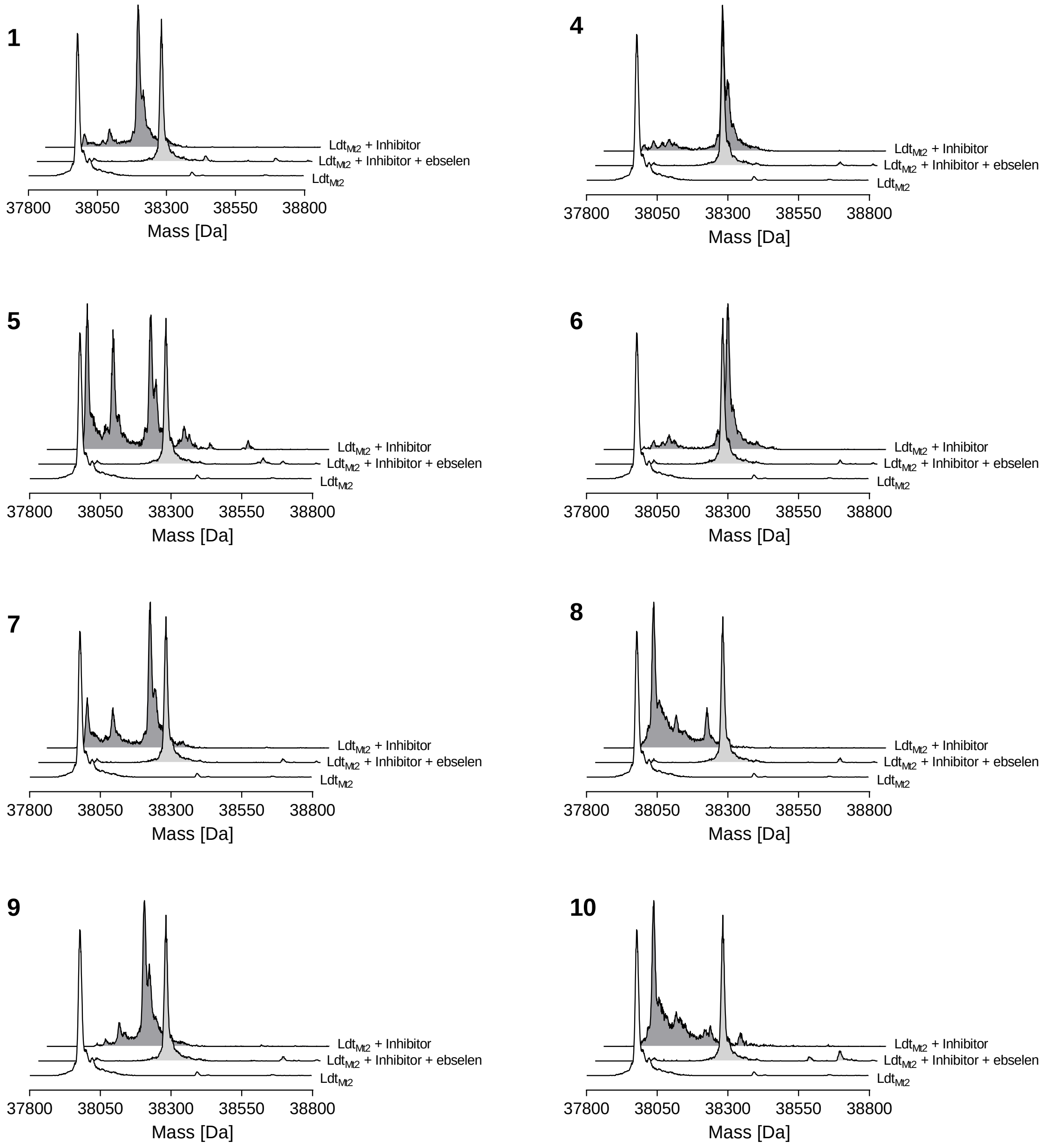

Figure S11. Protein-observed SPE-MS based Cys354 selectivity assays. Ldt_{Mt2} (1 µM) was preincubated with ebselen (a reported Ldt_{Mt2} inhibitor[8] which reacts with the nucleophilic Cys354, 10 µM) for 1 h in 50 mM Tris, pH 7.5. Inhibitors **1** and **4** – **10** (100 µM) were then added and samples were analysed after an additional 24 h incubation at room temperature using SPE-MS. The spectrum in white corresponds to Ldt_{Mt2} reacted with ebselen. The spectrum in light grey corresponds to Ldt_{Mt2} reacted with the specified inhibitor, following preincubation with ebselen. The spectrum in dark grey corresponds to Ldt_{Mt2} reacted with the specified inhibitor. Deconvoluted spectra, obtained using the maximum entropy algorithm in the MassHunter Workstation Qualitative Analysis B.07.00 program (Agilent), are shown.

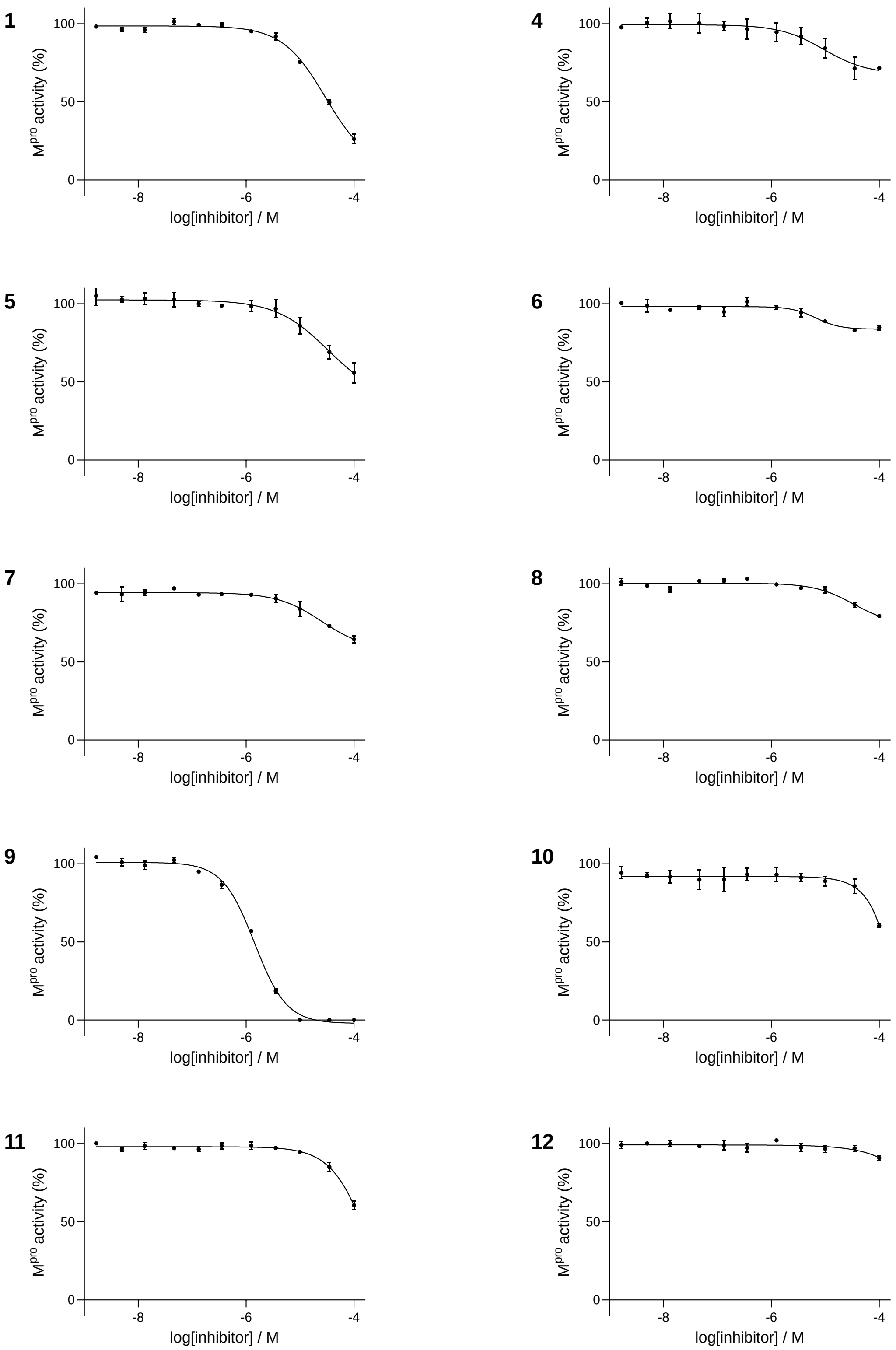

Figure S12. Representative dose-response curves for DEKs 1 and 4-11 and the mono-epoxide ketone 12 with SARS-CoV-2 M^{pro}. Inhibition assays were carried out using 150 nM M^{pro} and 2 µM 37mer peptide, with 15 min pre-incubation at rt in 20 mM HEPES, pH 7.5, 50 mM NaCl and M^{pro} activity was determined by SPE-MS.[5] Error bars represent the standard deviation of technical duplicates (n=2). Assays were performed in independent duplicates, each composed of technical duplicates; average pIC_{50} values and compound structures are given in Table S3.

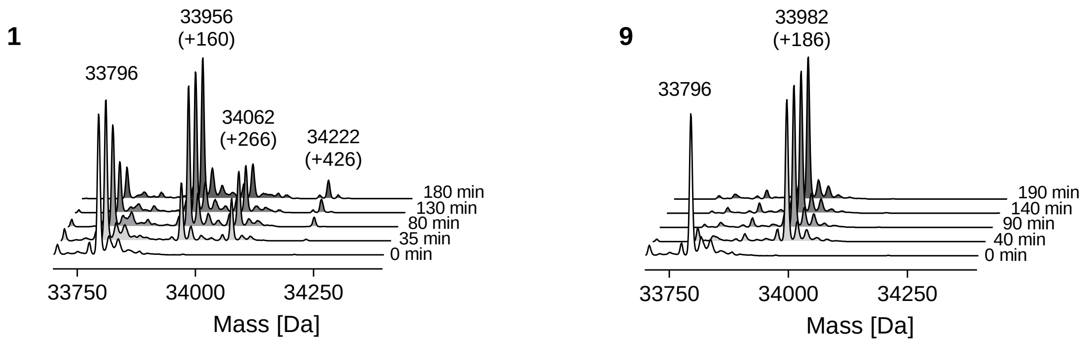

Figure S13. Protein observed SPE-MS analysis for the reaction of SARS-CoV-2 M^{pro} with DEKs 1 and 9. M^{pro} (2 µM) was incubated with DEKs **1** and **9** (20 µM) at rt in 20 mM HEPES, pH 7.5. Samples were analysed after the indicated times. Deconvoluted spectra, obtained using the maximum entropy algorithm in the MassHunter Workstation Qualitative Analysis B.07.00 program (Agilent), are shown. Mass shifts and assignments are analogous to those described in Table S1 for **1** and **9** for reaction with Ldt_{Mt2}.

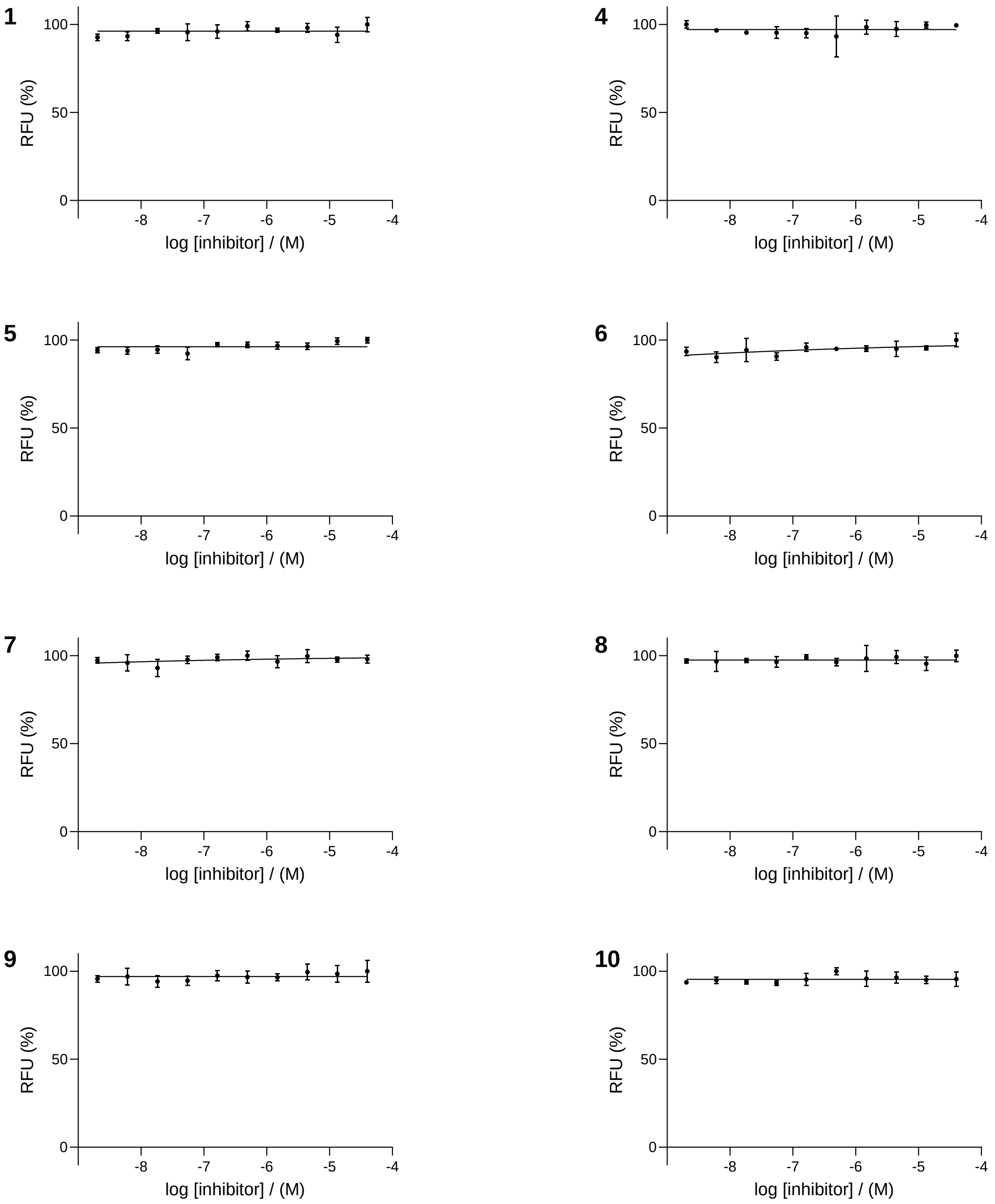

Figure S14. Dose-response curves for DEKs 1 and 4-10 with BlaC. Inhibition assays were carried out using 14 nM BlaC and 10 µM FC5[4] with a 10 min pre-incubation at rt in 100 mM sodium phosphate pH 7.5 with 0.01% (*v/v*) Triton X-100. Error bars represent the standard deviation (n=4). Average pIC_{50} values and compound structures are given in Table S3.

References

1. C. T. Lohans, H. T. H. Chan, T. R. Malla, K. Kumar, J. J. A. G. Kamps, D. J. B. McArdle, E. Van Groesen, M. de Munnik, C. L. Tooke, J. Spencer, R. S. Paton, J. Brem and C. J. Schofield, *Angew. Chem. Int. Ed.*, 2019, **58**, 1990-1994.
2. T. R. Malla, A. Tumber, T. John, L. Brewitz, C. Strain-Damerell, C. D. Owen, P. Lukacik, H. T. H. Chan, P. Maheswaran, E. Salah, F. Duarte, H. Yang, Z. Rao, M. A. Walsh and C. J. Schofield, *Chem. Commun.*, 2021, **57**, 1430-1433.
3. M. de Munnik, C. T. Lohans, G. W. Langley, C. Bon, J. Brem and C. J. Schofield, *ChemBioChem*, 2020, **21**, 368-372.
4. S. S. Van Berkel, J. Brem, A. M. Rydzik, R. Salimraj, R. Cain, A. Verma, R. J. Owens, C. W. G. Fishwick, J. Spencer and C. J. Schofield, *J. Med. Chem.*, 2013, **56**, 6945-6953.
5. T. R. Malla, L. Brewitz, D.-G. Muntean, H. Aslam, C. D. Owen, E. Salah, A. Tumber, P. Lukacik, C. Strain-Damerell, H. Mikolajek, M. A. Walsh and C. J. Schofield, *J. Med. Chem.*, 2022, **65**, 7682-7696.
6. M. de Munnik, P. A. Lang, F. De Dios Antos, M. Cacho, R. H. Bates, J. Brem, B. Rodríguez-Miquel and C. J. Schofield, *Chem. Sci.*, 2023, **14**, 7262-7278.
7. A. J. McCoy, R. W. Grosse-Kunstleve, P. D. Adams, M. D. Winn, L. C. Storoni and R. J. Read, *J. Appl. Crystallogr.*, 2007, **40**, 658-674.
8. M. de Munnik, C. T. Lohans, P. A. Lang, G. W. Langley, T. R. Malla, A. Tumber, C. J. Schofield and J. Brem, *Chem. Commun.*, 2019, **55**, 10214-10217.
9. P. D. Adams, P. V. Afonine, G. Bunkóczi, V. B. Chen, I. W. Davis, N. Echols, J. J. Headd, L.-W. Hung, G. J. Kapral, R. W. Grosse-Kunstleve, A. J. McCoy, N. W. Moriarty, R. Oeffner, R. J. Read, D. C. Richardson, J. S. Richardson, T. C. Terwilliger and P. H. Zwart, *Acta Crystallogr. D*, 2010, **66**, 213-221.
10. P. Emsley, B. Lohkamp, W. G. Scott and K. Cowtan, *Acta Crystallogr. D*, 2010, **66**, 486-501.
11. D. Liebschner, P. V. Afonine, N. W. Moriarty, B. K. Poon, O. V. Sobolev, T. C. Terwilliger and P. D. Adams, *Acta Crystallogr. D*, 2017, **73**, 148-157.
12. L. Palatinus and G. Chapuis, *J. Appl. Crystallogr.*, 2007, **40**, 786-790.
13. P. Parois, R. I. Cooper and A. L. Thompson, *Chem. Cent. J.*, 2015, **9**.
14. R. I. Cooper, A. L. Thompson and D. J. Watkin, *J. Appl. Crystallogr.*, 2010, **43**, 1100-1107.
15. D. G. Gibson, L. Young, R.-Y. Chuang, J. C. Venter, C. A. Hutchison and H. O. Smith, *Nat. Methods*, 2009, **6**, 343-345.
16. A. Arnold, M. Markert and R. Mahrwald, *Synthesis*, 2006, DOI: 10.1055/s-2006-926346, 1099-1102.
17. W. M. Weber, L. A. Hunsaker, C. N. Roybal, E. V. Bobrovnikova-Marjon, S. F. Abcouwer, R. E. Royer, L. M. Deck and D. L. Vander Jagt, *Bioorg. Med. Chem.*, 2006, **14**, 2450-2461.
18. V. K. Yadav and K. K. Kapoor, *Tetrahedron*, 1996, **52**, 3659-3668.
19. US9174960B2, 2015.
20. K. D. Ashtekar, X. Ding, E. Toma, W. Sheng, H. Gholami, C. Rahn, P. Reed and B. Borhan, *Org. Lett.*, 2016, **18**, 3976-3979.
21. E. Aguilera, J. Varela, E. Birriel, E. Serna, S. Torres, G. Yaluff, N. V. De Bilbao, B. Aguirre-López, N. Cabrera, S. Díaz Mazariegos, M. T. De Gómez-Puyou, A. Gómez-Puyou, R. Pérez-Montfort, L. Minini, A. Merlino, H. Cerecetto, M. González and G. Alvarez, *ChemMedChem*, 2016, **11**, 1328-1338.
22. A. Hasaninejad, A. Zare, L. Balooty, H. Mehregan and M. Shekouhy, *Synth. Commun.*, 2010, **40**, 3488-3495.
23. W. M. Weber, L. A. Hunsaker, S. F. Abcouwer, L. M. Deck and D. L. Vander Jagt, *Bioorg. Med. Chem.*, 2005, **13**, 3811-3820.

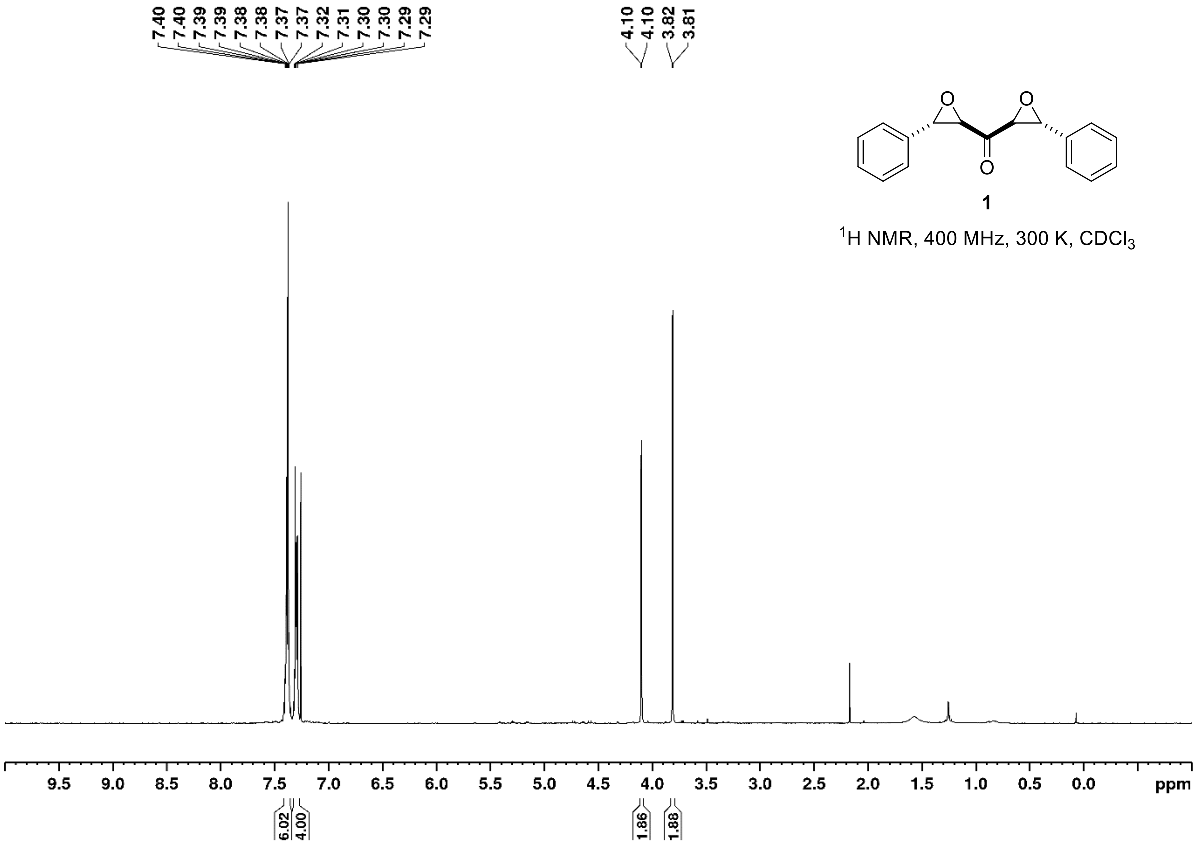

1

^{1}H NMR, 400 MHz, 300 K, $CDCl_3$

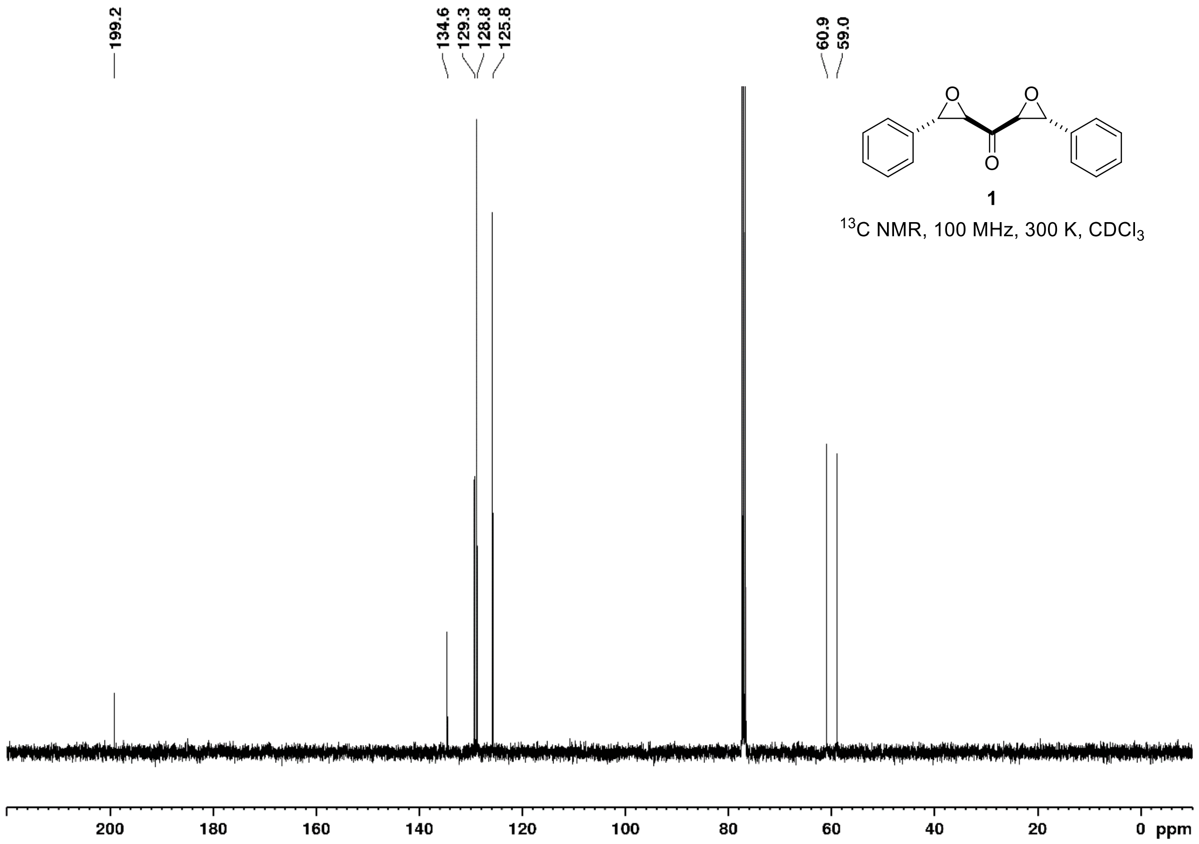

1

^{13}C NMR, 100 MHz, 300 K, $CDCl_3$

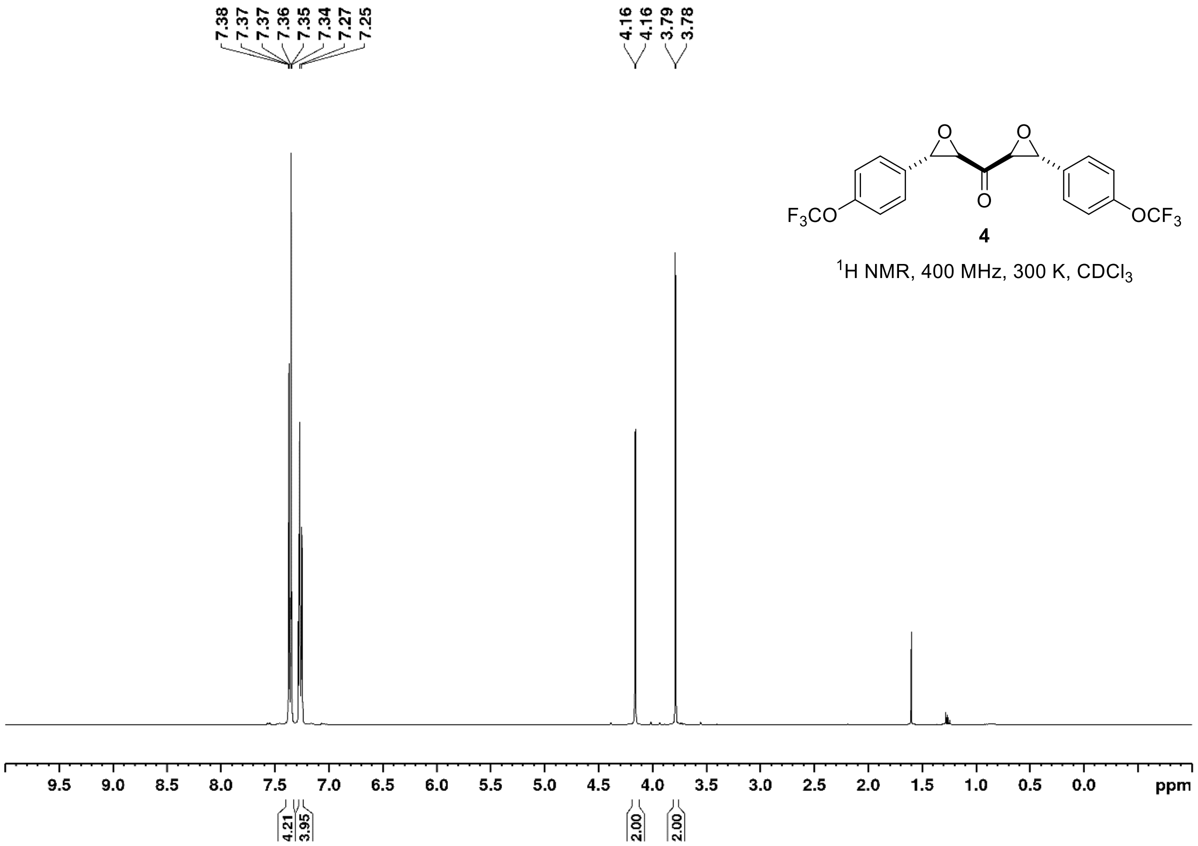
7.38
7.37
7.37
7.36
7.35
7.34
7.27
7.25
4.16
4.16
3.79
3.78
F3CO
OCF3
4
1H NMR, 400 MHz, 300 K, CDCl3
9.5 9.0 8.5 8.0 7.5 7.0 6.5 6.0 5.5 5.0 4.5 4.0 3.5 3.0 2.5 2.0 1.5 1.0 0.5 0.0 ppm
4.21
3.95
2.00
2.00

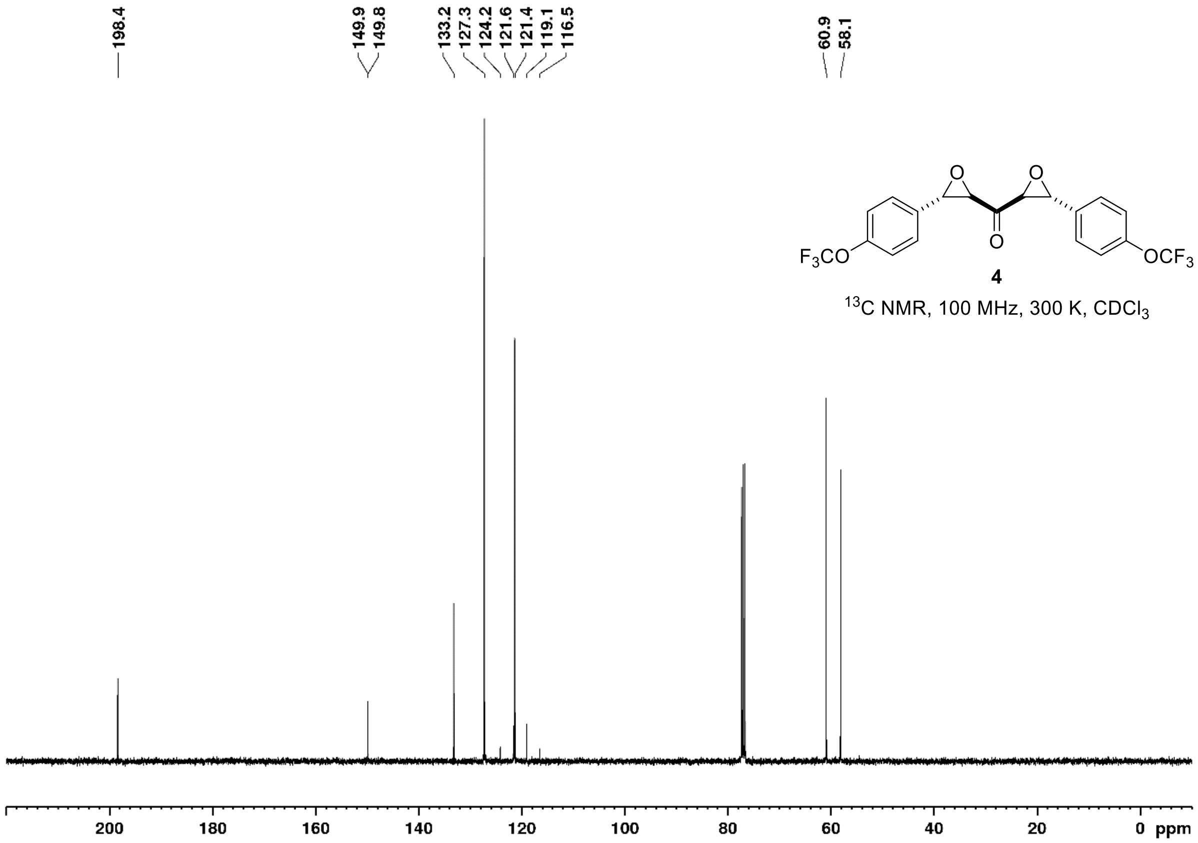
198.4
149.9
149.8
133.2
127.3
124.2
121.6
121.4
119.1
116.5
60.9
58.1
F3CO
OCF3
4
13C NMR, 100 MHz, 300 K, CDCl3
200 180 160 140 120 100 80 60 40 20 0 ppm

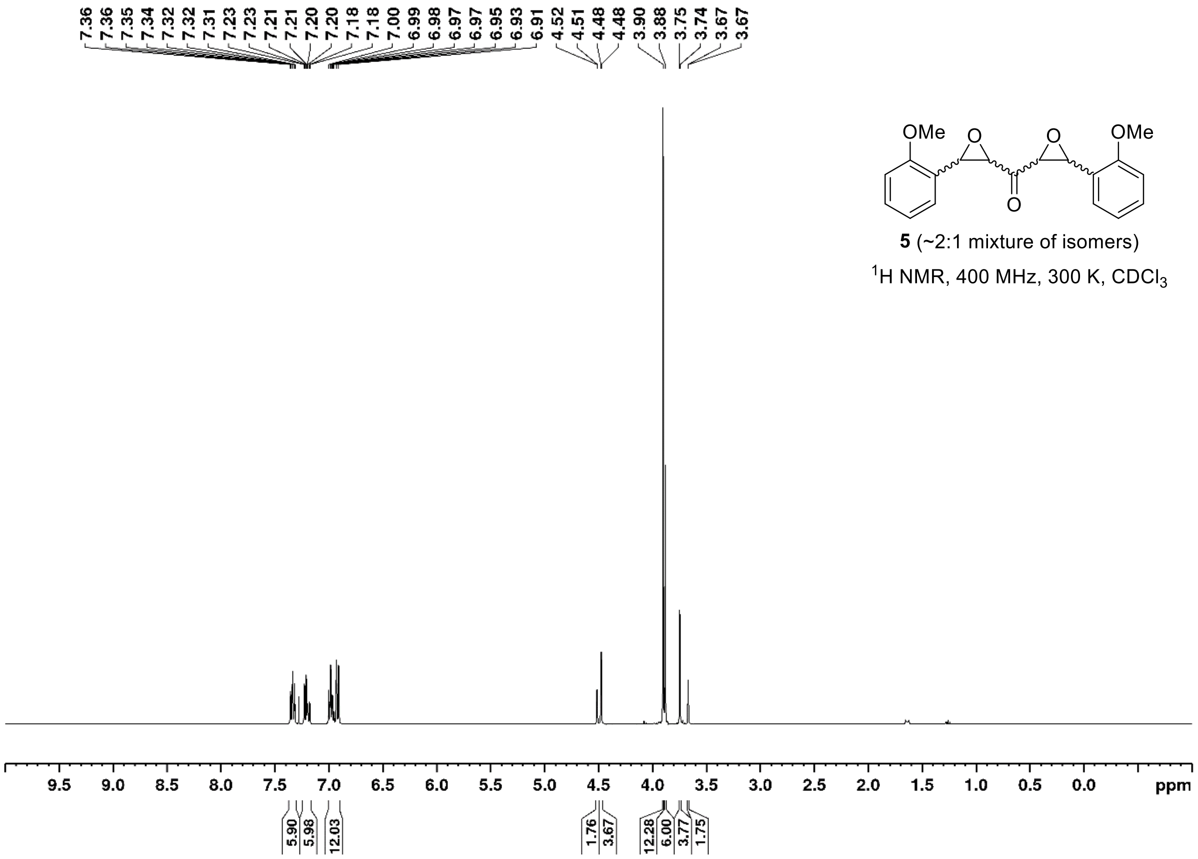
7.36 7.36 7.35 7.34 7.32 7.32 7.31 7.23 7.23 7.21 7.21 7.20 7.20 7.18 7.18 7.00 6.99 6.98 6.97 6.97 6.95 6.93 6.91 4.52 4.51 4.48 4.48 3.90 3.88 3.75 3.74 3.67 3.67
OMe O O OMe
O
5 (~2:1 mixture of isomers)
^{1}H NMR, 400 MHz, 300 K, $CDCl_3$
9.5 9.0 8.5 8.0 7.5 7.0 6.5 6.0 5.5 5.0 4.5 4.0 3.5 3.0 2.5 2.0 1.5 1.0 0.5 0.0 ppm
5.90 5.98 12.03 1.76 3.67 12.28 6.00 3.77 1.75

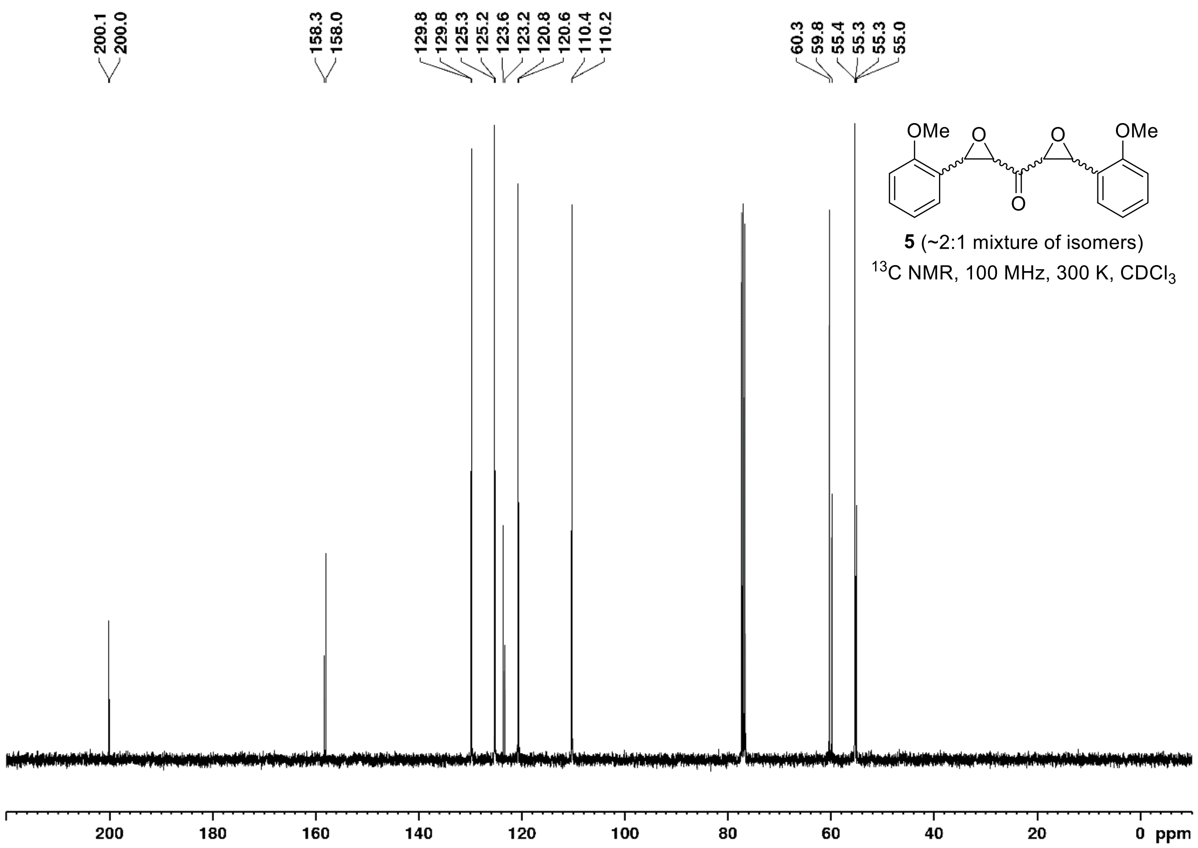
200.1 200.0 158.3 158.0 129.8 129.8 125.3 125.2 123.6 123.2 120.8 120.6 110.4 110.2 60.3 59.8 55.4 55.3 55.3 55.0
OMe O O OMe
O
5 (~2:1 mixture of isomers)
^{13}C NMR, 100 MHz, 300 K, $CDCl_3$
200 180 160 140 120 100 80 60 40 20 0 ppm

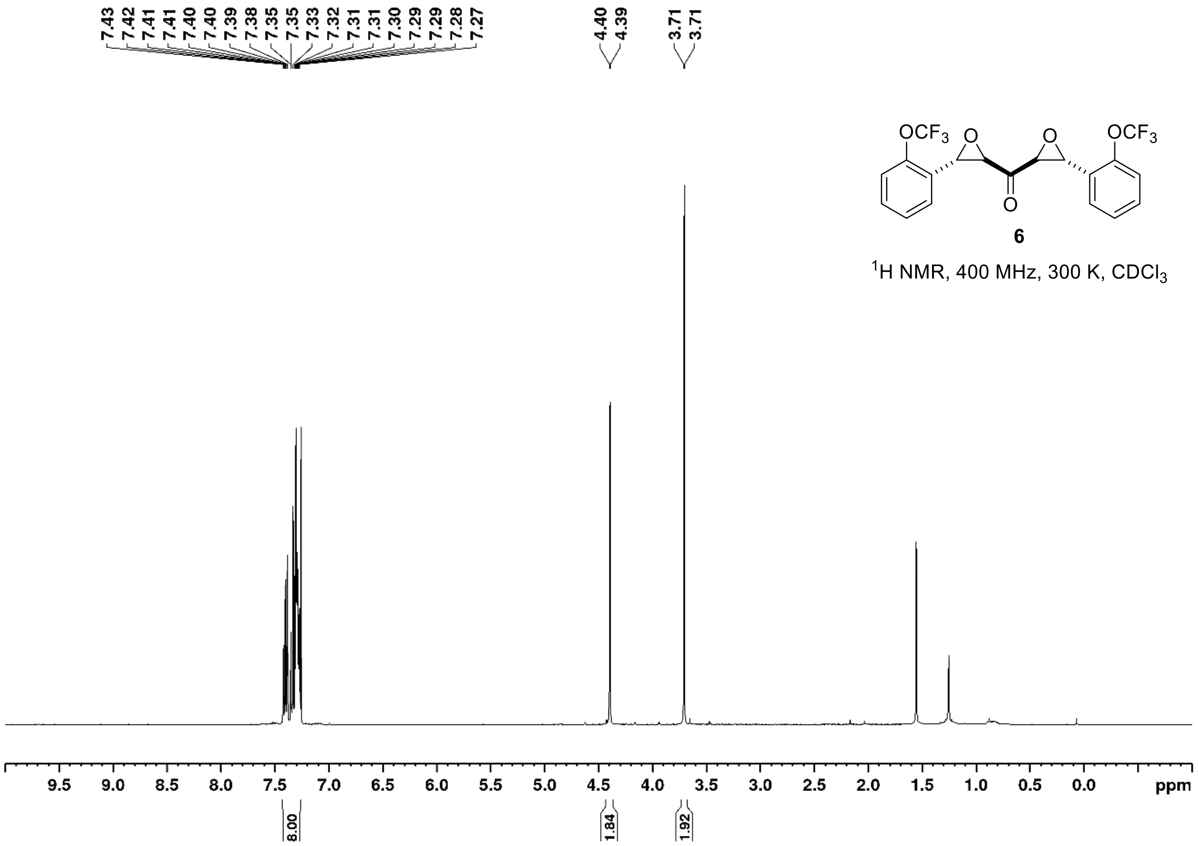

7.43 7.42 7.41 7.41 7.40 7.39 7.38 7.35 7.35 7.33 7.32 7.31 7.31 7.30 7.29 7.29 7.28 7.27
4.40 4.39
3.71 3.71
OCF3 O O OCF3
O
6
1H NMR, 400 MHz, 300 K, CDCl3
9.5 9.0 8.5 8.0 7.5 7.0 6.5 6.0 5.5 5.0 4.5 4.0 3.5 3.0 2.5 2.0 1.5 1.0 0.5 0.0 ppm
8.00
1.84
1.92

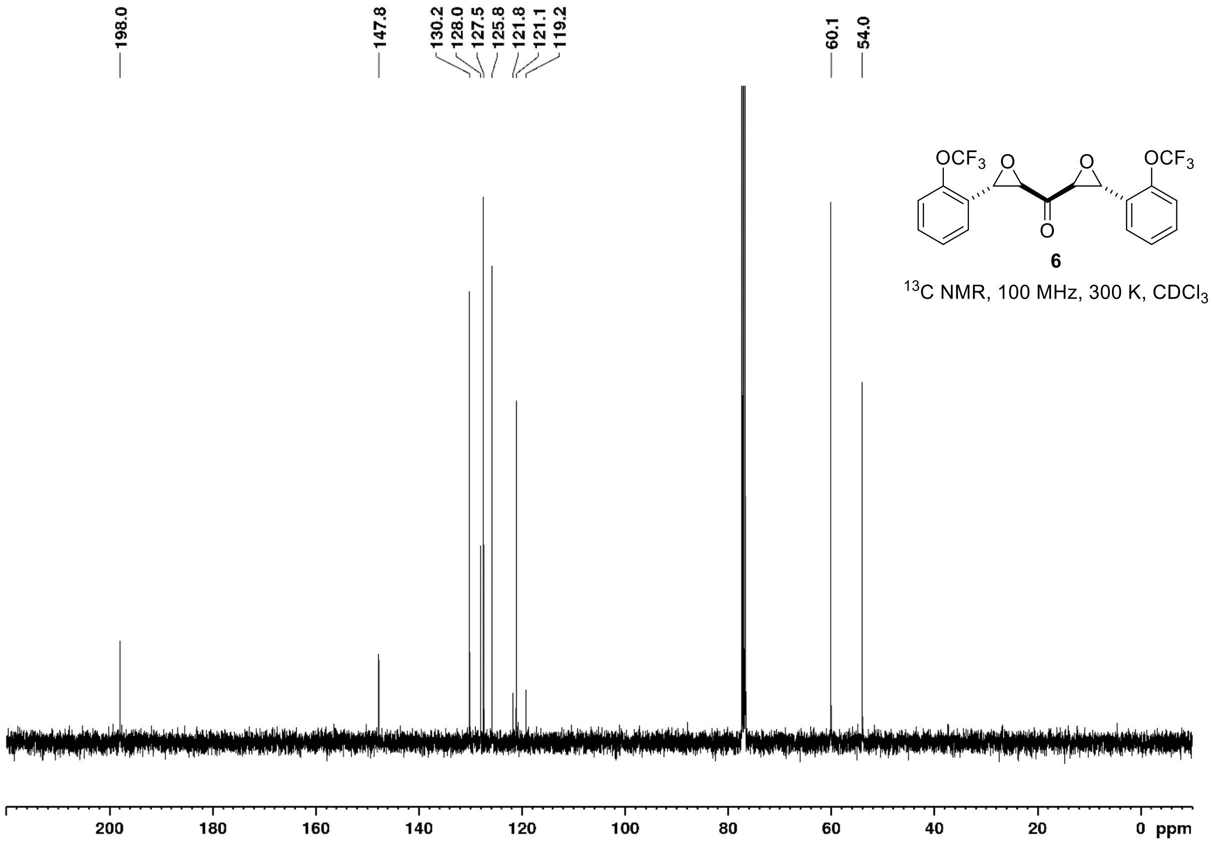

198.0
147.8
130.2 128.0 127.5 125.8 121.8 121.1 119.2
60.1
54.0
OCF3 O O OCF3
O
6
13C NMR, 100 MHz, 300 K, CDCl3
200 180 160 140 120 100 80 60 40 20 0 ppm

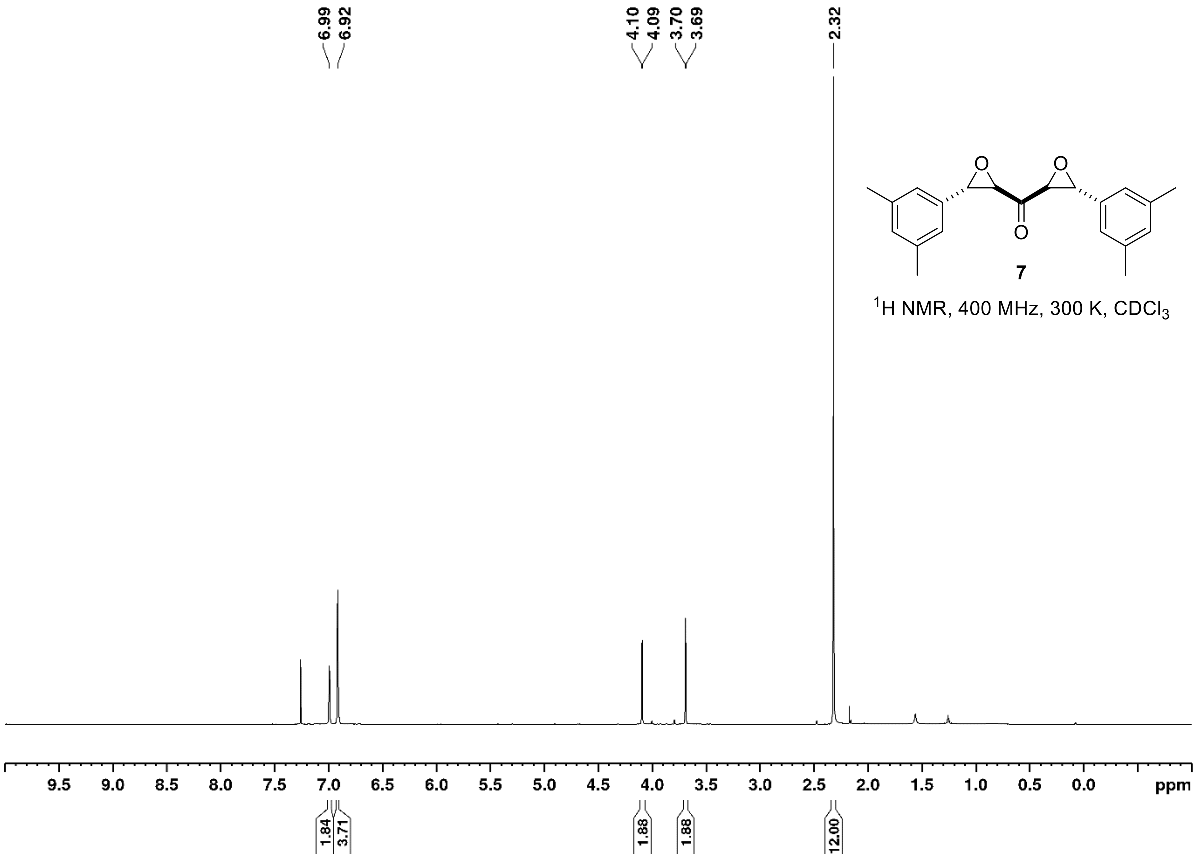
6.99
6.92
4.10
4.09
3.70
3.69
2.32
7
^{1}H NMR, 400 MHz, 300 K, $CDCl_3$
9.5 9.0 8.5 8.0 7.5 7.0 6.5 6.0 5.5 5.0 4.5 4.0 3.5 3.0 2.5 2.0 1.5 1.0 0.5 0.0 ppm
1.84
3.71
1.88
1.88
12.00

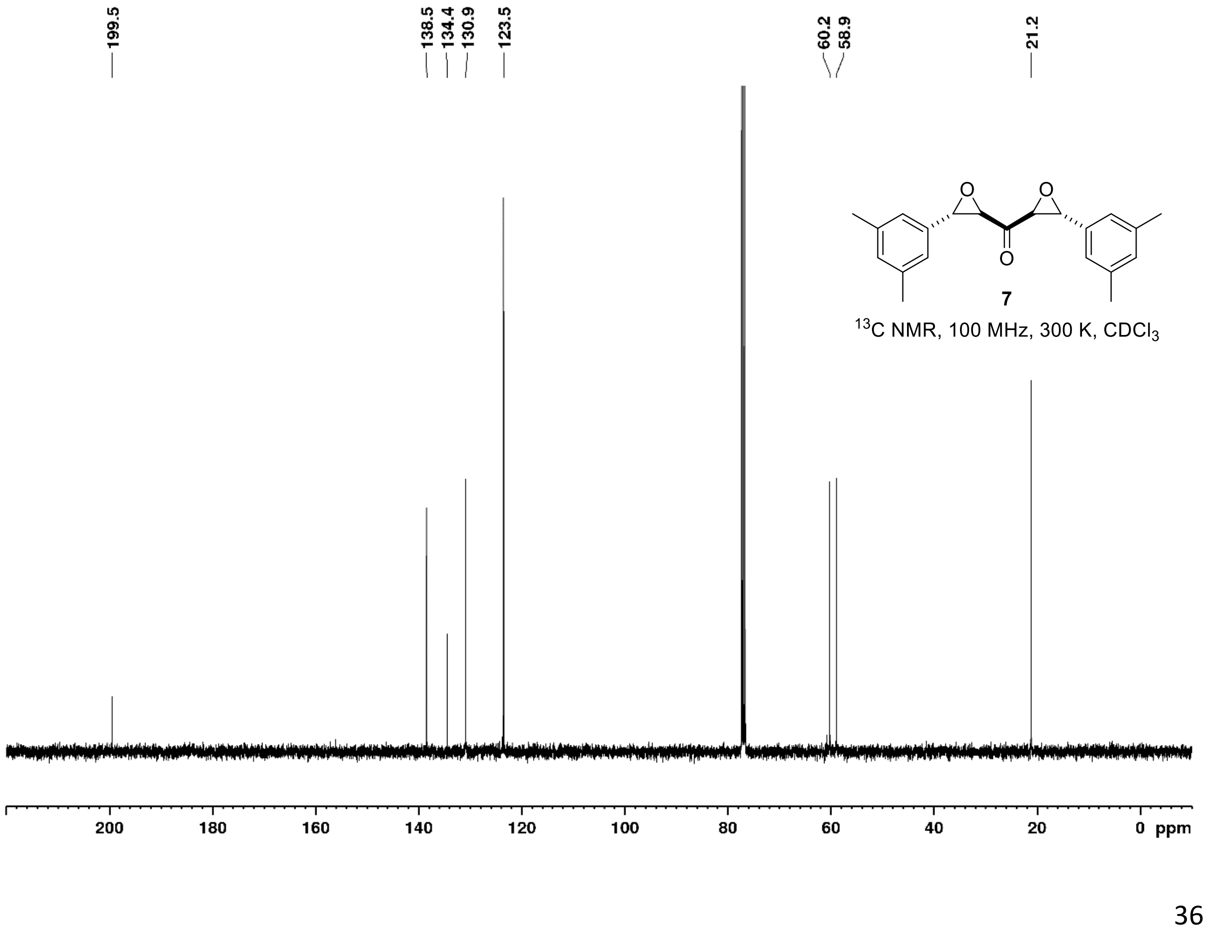
199.5
138.5
134.4
130.9
123.5
60.2
58.9
21.2
7
^{13}C NMR, 100 MHz, 300 K, $CDCl_3$
200 180 160 140 120 100 80 60 40 20 0 ppm

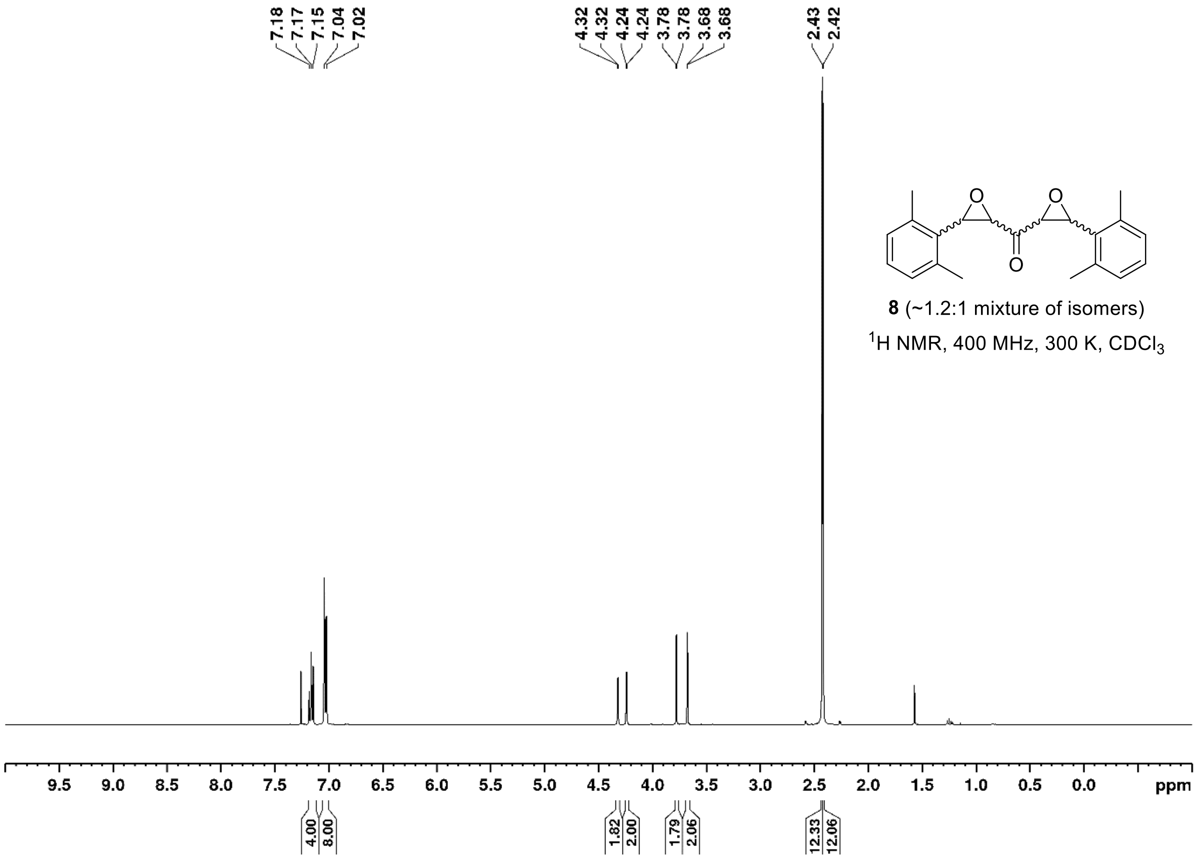
7.18
7.17
7.15
7.04
7.02
4.32
4.32
4.24
4.24
3.78
3.78
3.68
3.68
2.43
2.42
O
O
O
8 (~1.2:1 mixture of isomers)
1H NMR, 400 MHz, 300 K, CDCl3
9.5 9.0 8.5 8.0 7.5 7.0 6.5 6.0 5.5 5.0 4.5 4.0 3.5 3.0 2.5 2.0 1.5 1.0 0.5 0.0 ppm
4.00
8.00
1.82
2.00
1.79
2.06
12.33
12.06

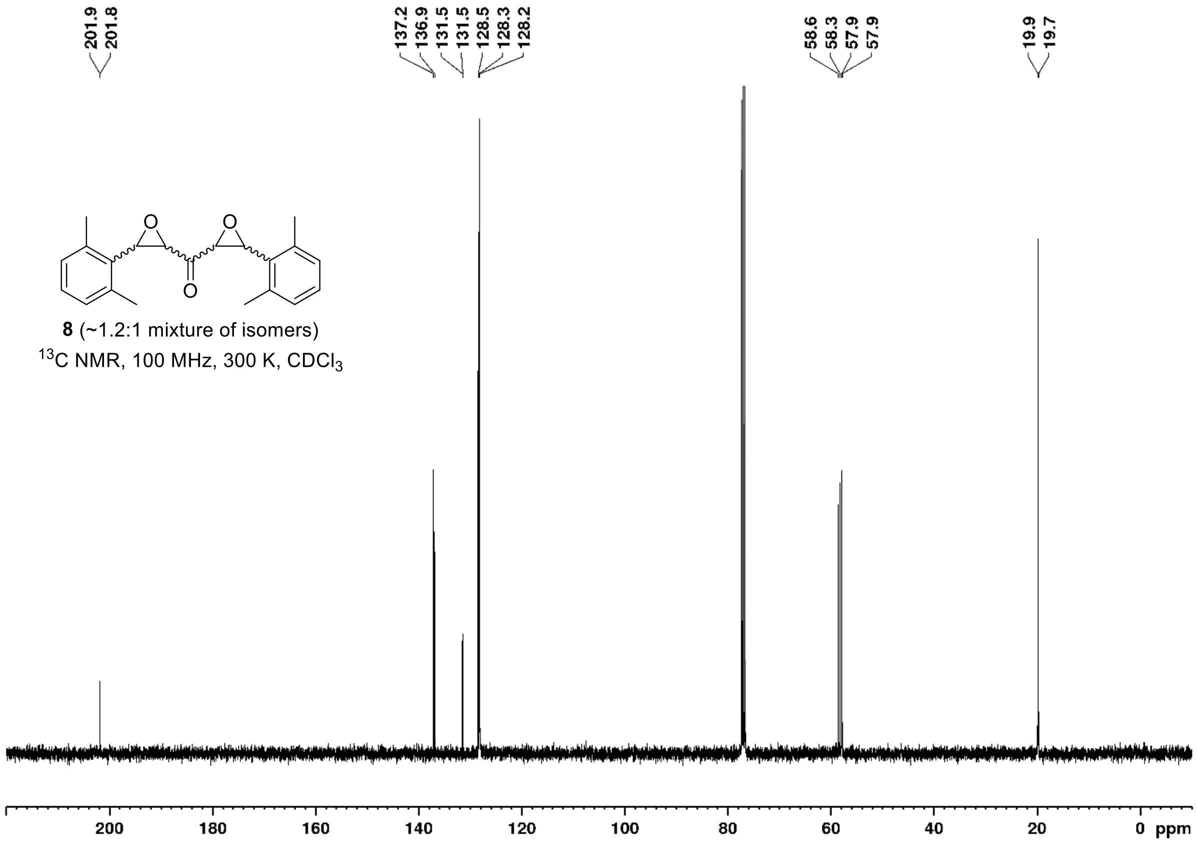
201.9
201.8
137.2
136.9
131.5
131.5
128.5
128.3
128.2
58.6
58.3
57.9
57.9
19.9
19.7
O
O
O
8 (~1.2:1 mixture of isomers)
13C NMR, 100 MHz, 300 K, CDCl3
200 180 160 140 120 100 80 60 40 20 0 ppm

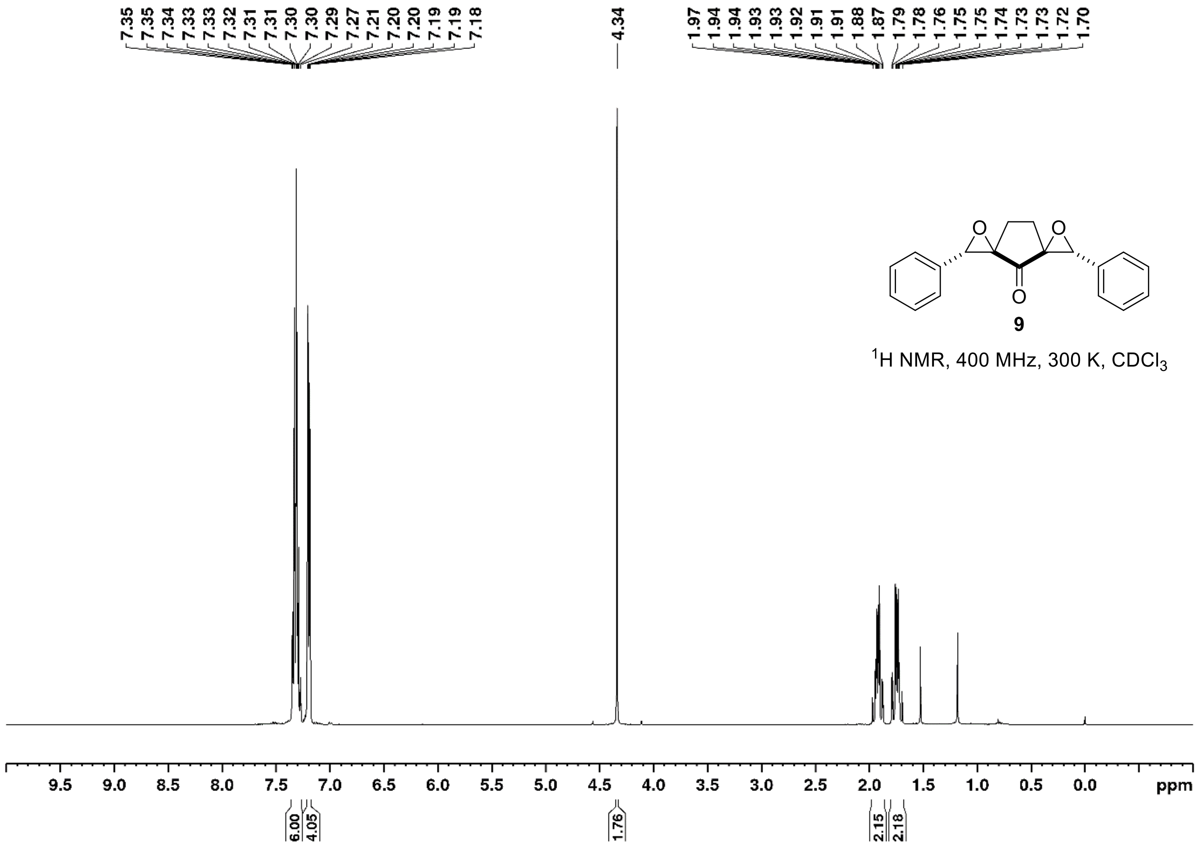
9
^{1}H NMR, 400 MHz, 300 K, CDCl$_3$
ppm

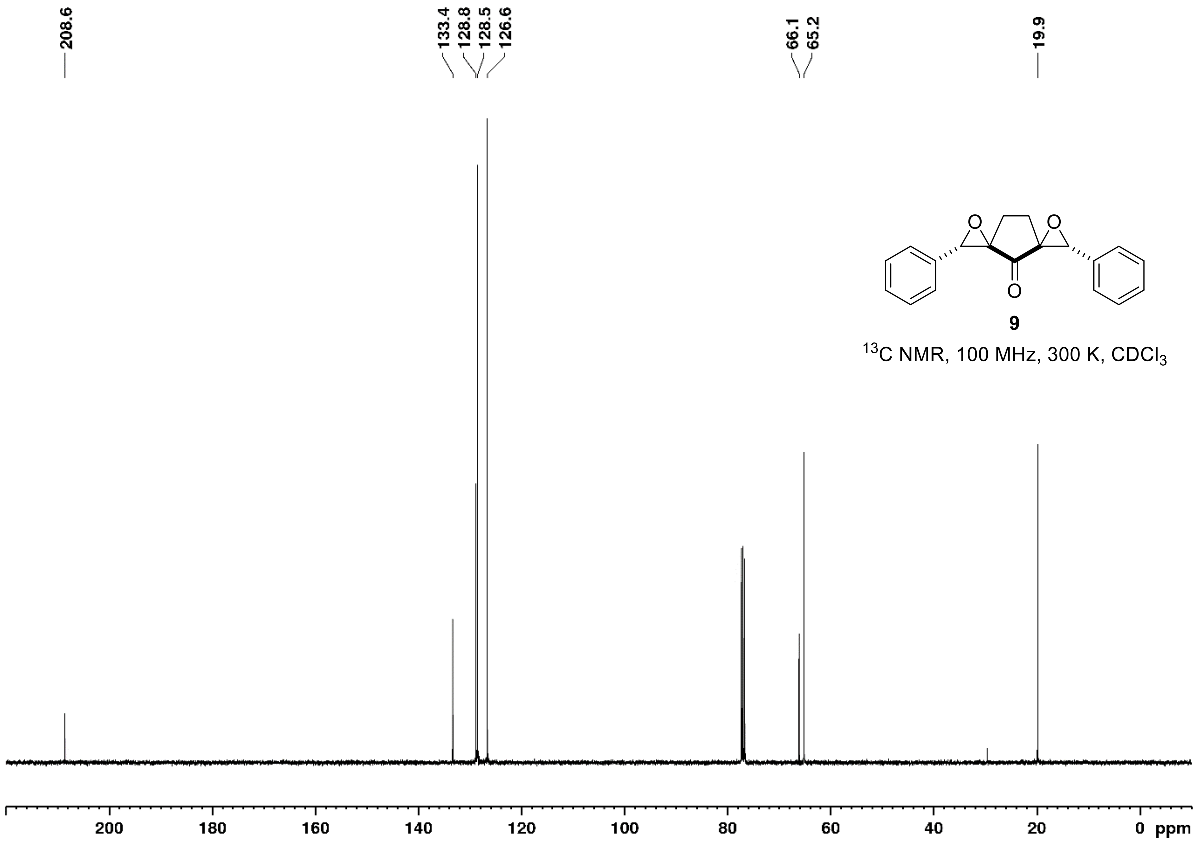
9
^{13}C NMR, 100 MHz, 300 K, CDCl$_3$
ppm

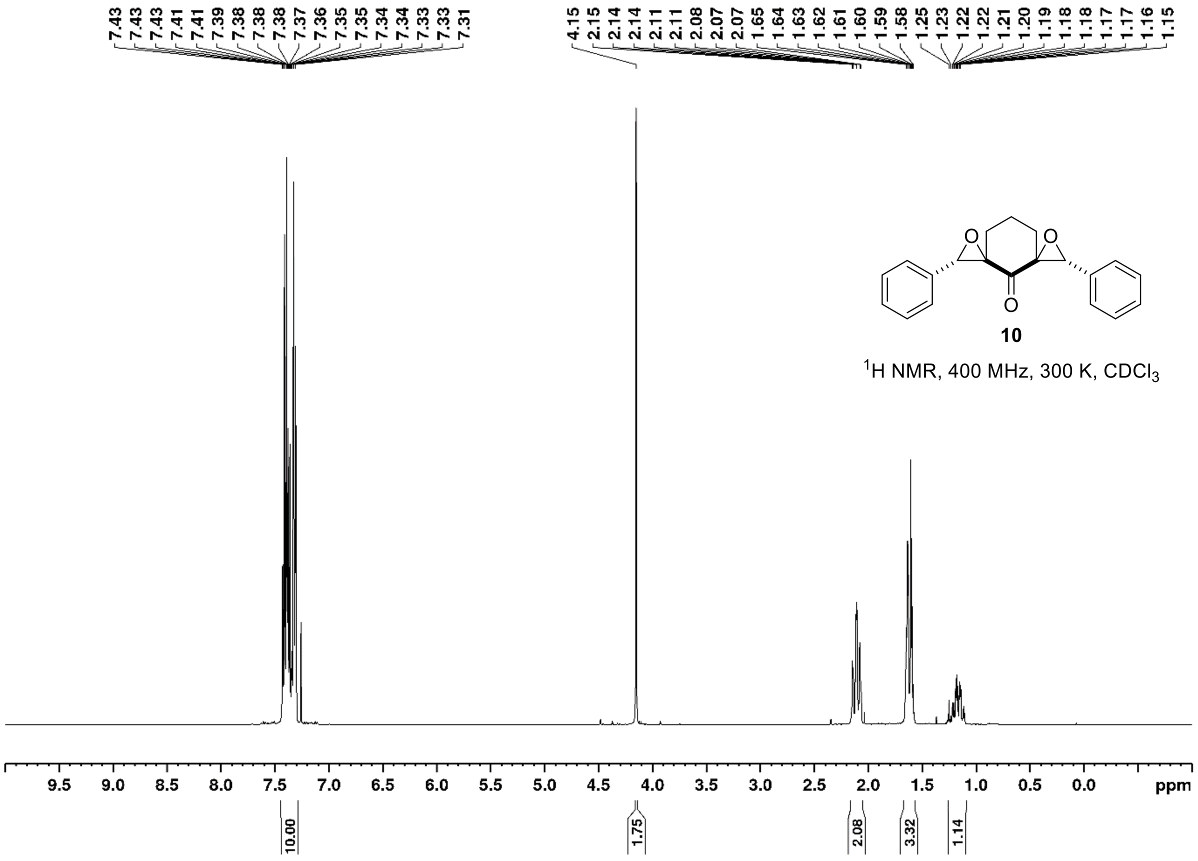
7.43 7.43 7.41 7.41 7.39 7.38 7.38 7.38 7.37 7.36 7.35 7.35 7.34 7.34 7.33 7.33 7.31
4.15 2.15 2.14 2.14 2.11 2.11 2.08 2.07 2.07 1.65 1.64 1.63 1.62 1.61 1.60 1.59 1.58 1.25 1.23 1.22 1.22 1.21 1.20 1.19 1.18 1.18 1.17 1.17 1.16 1.15
O
O
O
10
^{1}H NMR, 400 MHz, 300 K, $CDCl_3$
9.5 9.0 8.5 8.0 7.5 7.0 6.5 6.0 5.5 5.0 4.5 4.0 3.5 3.0 2.5 2.0 1.5 1.0 0.5 0.0 ppm
10.00
1.75
2.08
3.32
1.14

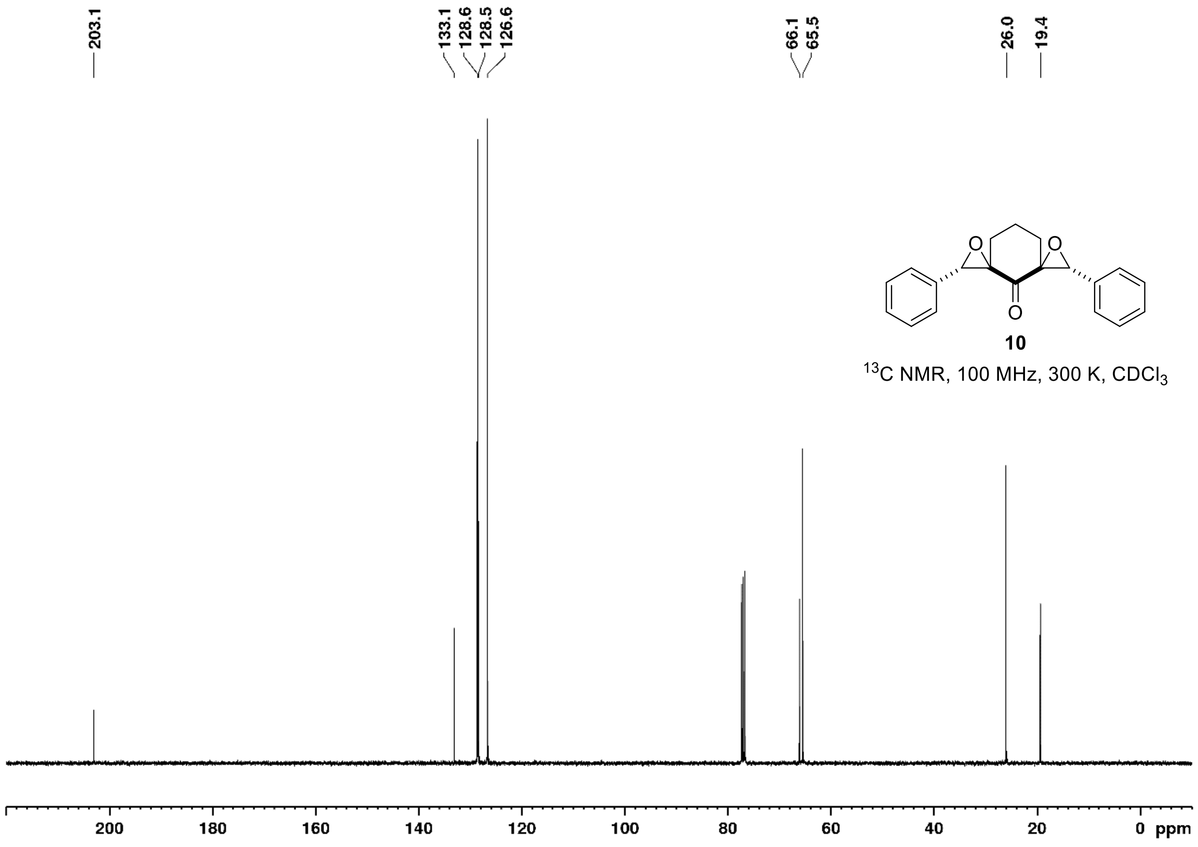
203.1
133.1 128.6 128.5 126.6
66.1 65.5
26.0 19.4
O
O
O
10
^{13}C NMR, 100 MHz, 300 K, $CDCl_3$
200 180 160 140 120 100 80 60 40 20 0 ppm

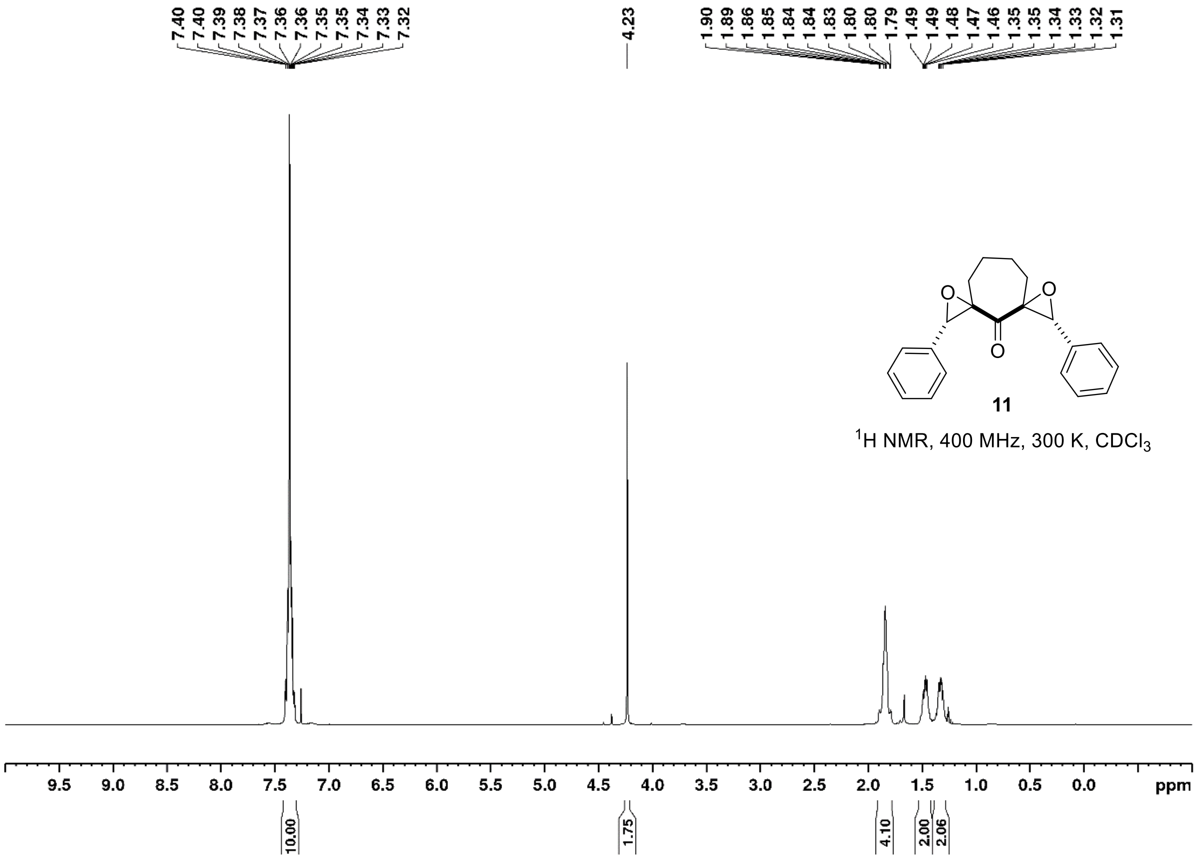
7.40 7.40 7.39 7.38 7.37 7.36 7.36 7.35 7.35 7.34 7.33 7.32
4.23
1.90 1.89 1.86 1.85 1.84 1.84 1.83 1.80 1.80 1.79 1.49 1.49 1.48 1.47 1.46 1.35 1.35 1.34 1.33 1.32 1.31
11
1H NMR, 400 MHz, 300 K, CDCl3
9.5 9.0 8.5 8.0 7.5 7.0 6.5 6.0 5.5 5.0 4.5 4.0 3.5 3.0 2.5 2.0 1.5 1.0 0.5 0.0 ppm
10.00 1.75 4.10 2.00 2.06

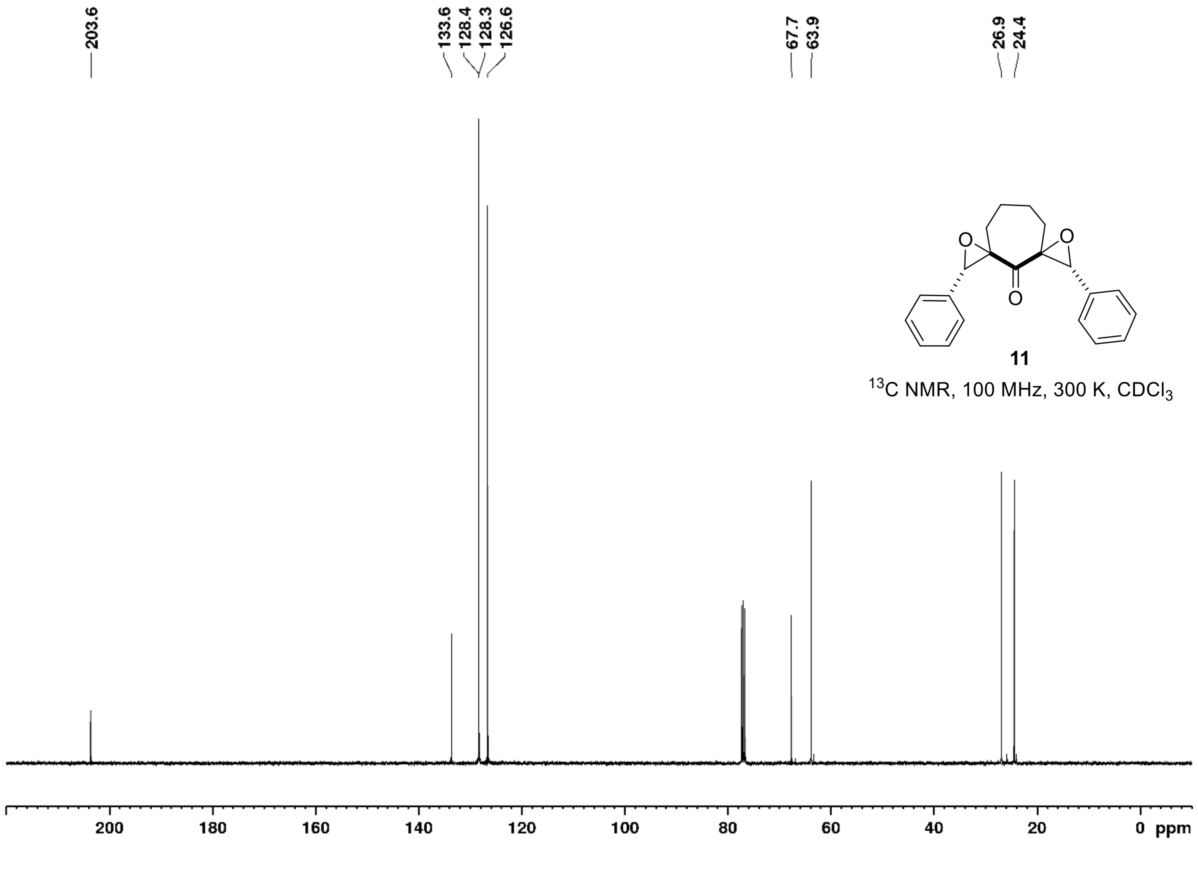
203.6
133.6 128.4 128.3 126.6
67.7 63.9
26.9 24.4
11
13C NMR, 100 MHz, 300 K, CDCl3
200 180 160 140 120 100 80 60 40 20 0 ppm
